# Efficacy of thymosin α1 for sepsis: a systematic review and meta-analysis of randomized controlled trials

**DOI:** 10.3389/fcimb.2025.1673959

**Published:** 2025-09-03

**Authors:** Bin Gu, Yu Zhou, Yao Nie, Luhao Wang, Liqun Liang, Zihuai Liao, Jingyi Wen, Xiangdong Guan, Minying Chen, Jianfeng Wu, Fei Pei

**Affiliations:** ^1^ Department of Critical Care Medicine, The First Affiliated Hospital, Sun Yat-sen University, Guangzhou, Guangdong, China; ^2^ Guangdong Clinical Research Center for Critical Care Medicine, Guangzhou, Guangdong, China

**Keywords:** sepsis, thymosin α1, personalized immunotherapy, heterogeneity of treatment effects, trial sequential analysis

## Abstract

**Background:**

Despite advances in understanding sepsis pathophysiology and extensive research, few treatments effectively target its underlying immune dysfunction. Thymosin α1 (Tα1) shows promise as an immunomodulator, but its impact on sepsis remains unclear.

**Methods:**

A search strategy was designed to include any prospective clinical studies using Tα1 for assessing 28-day mortality in patients with sepsis, excluding combination therapy studies. We conducted trial sequential analysis (TSA) to assess the robustness of meta-analyses findings. Heterogeneity of treatment effects (HTE) was conducted based on individual data from two multicenter randomized clinical trials (RCTs), with result credibility assessed through the instrument to assess the credibility of effect modification analyses (ICEMAN).

**Results:**

Out of 3003 identified studies, 11 RCTs met the inclusion criteria (967 patients in Tα1 group and 960 patients in control group). The comprehensive meta-analysis demonstrated a significant reduction in 28-day mortality associated with Tα1 administration (OR 0.73, 95%CI: 0.59-0.90, *P* = 0.003). Nonetheless, analyses of high-quality (OR 0.82, 95%CI: 0.65-1.03, *P* = 0.09) and multi-center (OR 0.86, 95%CI: 0.68-1.08, *P* = 0.20) subgroups did not reveal a mortality benefit. The HTE analysis of multiple subgroups in two large RCTs (representing 75% of the total patients) showed heterogeneity. Potential benefits were noted in subgroups of cancer (moderate credibility), diabetes (low credibility), and coronary heart disease (low credibility). Furthermore, the trial sequential analysis (TSA) suggests that the current sample size is inadequate.

**Conclusion:**

Tα1 has the potential to decrease 28-day mortality rates in patients with sepsis; however, it is crucial to recognize that its efficacy differs among various subgroups. These observations underscore the significance of personalized immunotherapy strategies in forthcoming clinical trials.

**Systematic review registration:**

https://www.crd.york.ac.uk/prospero/, identifier CRD42024628937.

## Introduction

Sepsis is defined as life-threatening organ dysfunction resulting from a dysregulated host response to infection, accounting for almost 20% of all deaths worldwide (Singer et al., 2016; Rudd et al., 2020; Meyer and Prescott, 2024). Dysregulated immune responses serve as critical intermediary between infection and organ dysfunction (van der Poll et al., 2017; Venet and Monneret, 2018; Hotchkiss and Opal, 2020). Immunomodulatory therapy is regarded as one of the most promising approaches to reduce mortality by regulating immune function in sepsis patients (Pei et al., 2018; Torres et al., 2022; Slim et al., 2024; Zhang et al., 2024).

Over 700 studies have investigated various immunomodulatory treatments, including Thymosin α1 (Tα1), granulocyte-macrophage colony-stimulating factor, and interleukin-7 (Slim et al., 2024). Of these, Tα1 is a promising drug with bidirectional modulatory function, exerting multiple effects in infectious diseases, such as promoting naive T cell maturation (Shehadeh et al., 2022), reversing T cell exhaustion (Liu et al., 2020), alleviating cytokine storms (Matteucci et al., 2021; Tian et al., 2025), and enhancing Th1-dependent antifungal immunity (Romani et al., 2004). A previous meta-analysis of 19 studies involving 1354 adult patients suggested that Tα1 might benefit patients with sepsis (Liu F. et al., 2016), However, the subsequent TESTS trial, the largest multicenter, double-blind RCT to date with 1106 patients with sepsis, found no mortality reduction or clinical improvement with Tα1, though elderly and diabetic subgroups showed potential effects (Wu et al., 2025), While the TESTS findings may influence practice, a comprehensive analysis of all available data is needed to establish definitive clinical guidance.

Traditional meta-analyses have predominantly concentrated on data pooling without adequately assessing statistical power. Trial sequential analysis (TSA) addressed this limitation by identifying type I and type II errors, thereby enhancing the reliability of meta-analytic findings (Shah and Smith, 2020). TSA also determines the necessary sample sizes for achieving meaningful outcomes and assesses the potential value of future trials. Furthermore, existing meta-analyses often neglect patient-centered outcomes, which constrains our understanding of how individual patient characteristics influence the efficacy of immunomodulatory therapy in sepsis, often erroneously implying a “one size fits all” approach.

Given these considerations, there is an urgent need for an updated meta-analysis to achieve the following objectives: (1) to synthesize the evidence regarding the efficacy of Tα1 in patients with sepsis overall, and (2) to evaluate the comparative efficacy of Tα1 across various subgroups of septic patients.

## Methods

### Study design and registration

This is an updated systematic review and meta-analysis aimed to evaluate the efficacy of adding Tα1 therapy compared to conventional therapy alone in reducing the 28-day mortality in patients with sepsis. We followed the PRISMA (Preferred Reporting Items for Systematic Reviews and Meta-Analyses) 2020 statement (Page et al., 2021). The protocol was registered at PROSPERO International prospective register of systematic reviews, with registration number CRD42024628937.

### Searching strategy

We searched for all studies that investigate whether Tα1 could improve the prognosis of sepsis or septic shock patients while excluding COVID-19 pandemic influences. Search terms “thymosin alpha1” or “thymosin” or “thymus” or “Maipuxin” or “thymalfasin” or “Zadaxin” referred to thymosin alpha1 and “severe infection” or “sepsis” or “septic shock” referred to sepsis were used. The strategy was implemented in both English and Chinese databases including PubMed, all databases of Web of Science, Embase, Cochrane library, China National Knowledge Internet (CNKI), China Science and Technology Journal Database (VIP) and Wanfang Database. Registers, websites, organizations, reference lists, preprints, conference literature and other sources were consulted to identify studies comprehensively. Searching strategies in each database were shown in **Supplementary Material**-search strategy.

The literature search was performed on December 4, 2024 and repeated on January 17, 2025 before final analysis. All the results were imported into EndNote X9 (Clarivate Analytics) software for further selection.

### Selection criteria

Inclusion criteria were as follows: (1) adults patients aged over 18 years; (2) reported the 28-day mortality; (3) according to the latest diagnostic criteria, the patient had to be diagnosed with sepsis, severe sepsis or septic shock; (4) Tα1 was the only different treatment in interference group; (5) patients in control group were treated with conventional therapy according to Surviving Sepsis Campaign (SSC) guidelines. The follows were excluded: (1) a review, case report or only abstract; (2) objects were animals or cells; (3) not provided related outcomes; (4) studies had not been completed. (5) key results cannot be extracted; (6) study about COVID-19.

One independent reviewer (BG) evaluated titles and abstracts while two reviewers (YZ and YN) thereafter screened full-text independently. References of the selected studies were also screened by the two reviewers afterwards. Duplicates were removed automatically and then manually using EndNote X9 software. We determined whether the included studies were RCTs or retrospective studies based on the descriptions in the abstracts and methods sections of each article. Disagreement was resolved by discussion with a third reviewer (FP).

### Data extraction

Two reviewers independently extracted data into consensual standard table. The following characteristics of included studies were collected: first author, publication year, type of study, implementation period, country and settings, dosage and time of Tα1 use, number of patients, primary and secondary outcomes. Besides, demographic characteristics of patients were also collected including age, gender, inclusion and exclusion criterion, biochemical indicators, 28-day mortality, ICU mortality, length of ICU stay, duration of mechanical ventilation, sequential organ failure assessment (SOFA) score and acute physiology and chronic health evaluation (APACHE) II score.

Any disagreement between the two reviewers was resolved by discussion or consulted with the third reviewer. When encountering self-contradictory data or errors in studies, we also e-mailed the author for more detailed information; if there was no response, the study was excluded.

### Quality assessment

The quality of studies included in extracting data process were judged by the Cochrane Collaboration’s tool for assessing risk of bias (Higgins et al., 2011). We used scores to quantify the quality of every study: for each aspect of bias, two scores for low risk of bias, one for unclear risk of bias and zero for high risk of bias (Georgiou et al., 2009; Fan et al., 2012). A total of 7 aspects of bias were judged and summed up. A maximum score of 14 was possible, with 11–14 considered relatively high quality and 0–10 considered relatively low quality. Two reviewers (YZ and BG) independently judged the studies. Disagreement was solved through discussion or consensus meeting with senior investigators. Any study that received a high score was reassessed.

### Statistical analysis

For dichotomous variables, odds ratio (OR) and 95% confidence intervals (CI) of every study were calculated. For continuous variables, weighted mean difference was calculated. I^2^ and chi-squared statistics were applied to estimate heterogeneity. The random-effects model was used if heterogeneity is significant (I^2^ ≥ 50%), otherwise the fixed-effects model was used (Higgins et al., 2003). Both the Mantel-Haenszel test and inverse-variance (I-V) weighting were applied.

We used Egger’s test to evaluate publication bias and constructed funnel plot if at least ten studies were available for meta-analysis (Sterne et al., 2011). The sensitivity analysis was conducted by taking each single study away from the total and reanalyzing the remaining studies. In addition, we performed the subgroup analyses to identify the source of heterogeneity and the effect of confounding based on the following variables: study quality, study design, dosage and applied SSC guidelines. The significance of the pooled index was determined using the Z test.

To mitigate the risk of misinterpreting random error in meta-analysis, trial sequential analysis (TSA) was performed for 28-day mortality using the TSA software (0.9.5.10 Beta, The Copenhagen Trial Unit, Denmark) (Shah and Smith, 2020). We set conventional test boundary at type I error 5% (two-sided), and dichotomous alpha-spending boundary using O’Brien-Fleming function at the same type I error. Information size was estimated through power of 90%, low bias based relative risk reduction (RRR) and calculated incidence in control group of all included randomized clinical trials (RCTs). Heterogeneity correction was model variance based.

We acquired detailed information to investigate hazard ratio (HR) of subgroups classified by age, gender, cancer, hypertension, diabetes, coronary heart disease and chronic obstructive pulmonary disease of two multi-center, high quality studies (Wu et al., 2013; Wu et al., 2025). Cox regression was used to calculate HR adjusted by different study with 95% CI as well as reporting test of interaction (Wallach et al., 2017). Results of subgroups with *P* < 0.1 for interaction were graded by Instrument for the Credibility of Effect Modification Analyses (ICEMAN) (Schandelmaier et al., 2020).

A two-tailed *P* < 0.05 was considered statistically significant. The meta-analysis was done using Stata/MP statistical software (version 14.0), Review Manager software (version 5.3) and SPSS software (version 25.0).

## Results

### Characteristics of eligible studies

**Figure 1:**
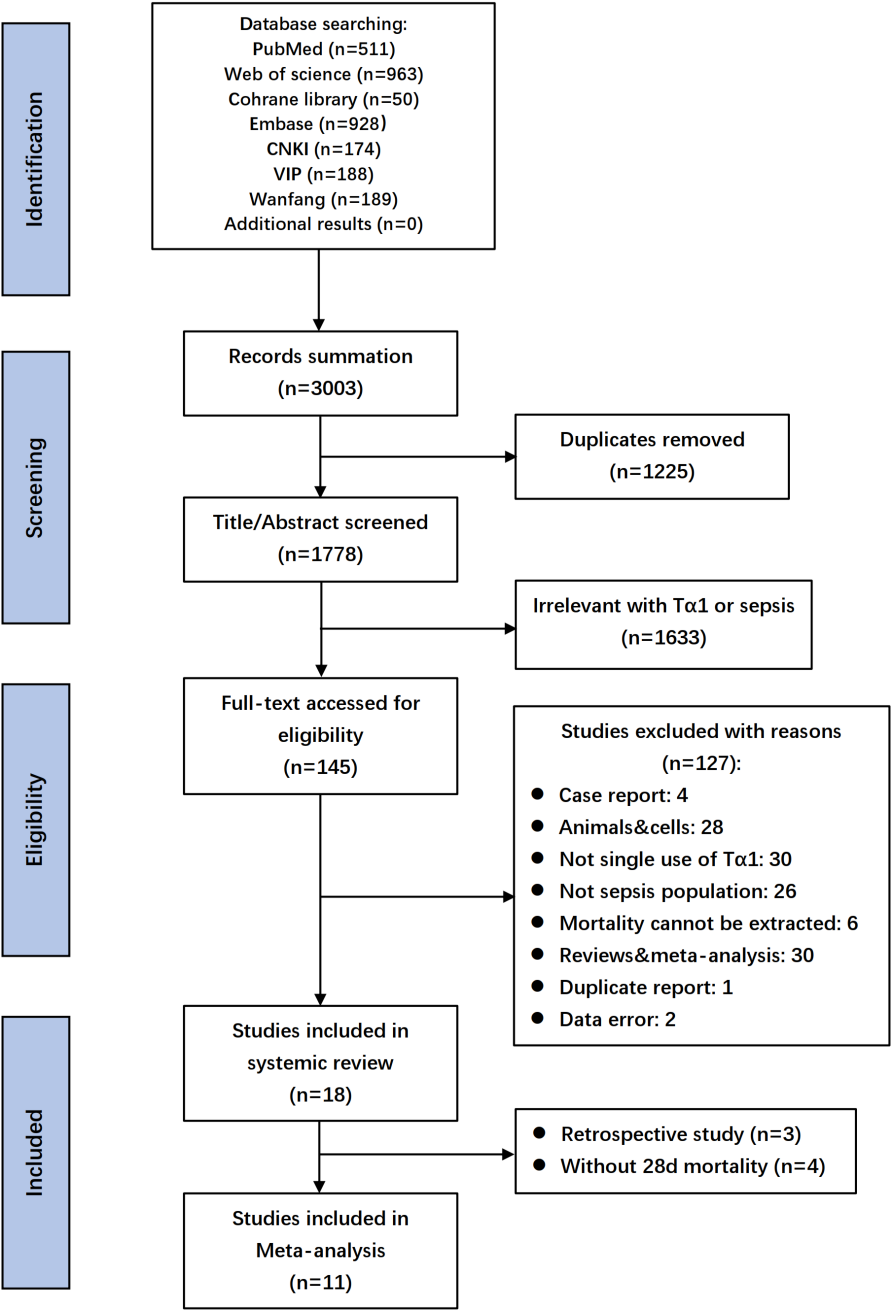
Flow diagram of searching and enrolling studies.

A database search identified 3003 records, with 18 selected after full-text screening. Excluding three retrospective studies and four RCTs lacking 28-day mortality data, 11 RCTs with 1927 patients remained for evaluating Tα1 efficacy in the meta-analysis (Wu and Fang, 2004; Chen, 2007; Zhou et al., 2009; Gui et al., 2012; Wu et al., 2013; Wu et al., 2014; Xiao et al., 2015; Hu et al., 2016; Pei et al., 2017; Yang et al., 2018; Wu et al., 2025). Flow diagram shown the details of screening process (**Figure 1**), and the characteristics of the included studies were listed in **Table 1** and **Supplementary Table S1**. Summary and details of quality assessment of each RCT were shown in the **Supplementary Figure S1**. Five studies scored 11 and above were regarded as relatively high quality.

**Table 1:** General characteristics of the 11 RCTs.

Study	Country	Implementation period	Study type	Study design	Total patients	Usage of Tα1	Observation and measurement
(Wu et al., 2014)	China	2002.7-2004.5	RCT	single-center	44	1.6mg every day for ten days	mHLA-DR, CRP, APACHE II, numbers of organ dysfunction, 28-day mortality
(Chen, 2007)	China	2000.5-2006.5	RCT	single-center	42	1.6mg twice a day for one week	T cells and NK cells counting, body temperature peak, length of ICU stay, mechanical ventilation, 28-day mortality
(Zhou et al., 2009)	China	2004.6-2007.10	RCT	single-center	47	1.6mg every day for one week	HLA-DR, T cells counting, APACHE II, Marshall scores, length ICU stay, mechanical ventilation, 28-day and 90-day mortality
(Gui et al., 2012)	China	2010.6-2011.2	RCT	multi-center	42	1.6mg every day for one week	Subsets of T cells, IgG, IgA, IgM, PCT, IL-1, IL-6, IL-10, APACHE II scores, 28-day mortality
(Wu et al., 2013)	China	2008.5-2010.12	RCT	multi-center	361	1.6mg twice a day for five days and then every for two days	28-day mortality, SOFA score, CD4/CD8, monocyte HLA-DR expression
(Wu et al., 2014)	China	2008.7-2009.8	RCT	single-center	54	1.6mg twice a day for five days and then every for two days	mRNA level of TLR2/TLR4/MYD88, 28-day mortality
(Xiao et al., 2015)	China	2013.10-2014.7	RCT	single-center	60	1.6mg every day for six days	Subsets of T cells, CD4/CD8, change of IgG, IgA and IgM, length of ICU stay, readmission to hospital, 28-day mortality
(Hu et al., 2016)	China	2012.7-2014.7	RCT	single-center	106	1.6mg twice a day for five days	HLA-DR, CD4/CD8, lymphocyte counting, WBC counting, cytokine, APACHE II, SOFA score, antibiotic usage, vasoactive agent, mechanical ventilation, length of ICU stay, ICU mortality, 28-day mortality
(Pei et al., 2017)	China	2016.3-2016.9	RCT	single-center	20	1.6mg twice a day for one week	SOFA score, PCT, CD86/PD-L1 expression of monocyte, length of ICU stay, 28-day mortality
(Yang et al., 2018)	China	2016.5-2017.6	RCT	single-center	62	1.6mg every day for one week	Subsets and apoptosis of lymphocytes, function of liver and kidney, APACHE II, length of ICU stay, 28-day mortality
(Wu et al., 2025)	China	2016.9-2021.3	RCT	multi-center	1089	1.6mg every 12 hours for one week	HLA-DR, Treg counting, NLR, length of ICU stay, organ supportive time, 28-day and 90-day mortality

RCT, randomized controlled trial. Tα1, thymosin alpha 1. SOFA, sequential organ failure assessment. APACHE, acute physiology and chronic health evaluation. CRP, C reaction protein. WBC, white blood cell. HLA-DR, human leukocyte antigen DR. TLR, toll-like receptor. MYD, myeloid differentiation factor. PBMC, peripheral blood mononuclear cell. PD-L1, programmed death ligand 1. TNF-α, tumor necrosis factor α. NLR, neutrophil to lymphocyte ratio. PCT, procalcitonin.

### Primary outcome for the 28-day mortality

**Figure 2:**
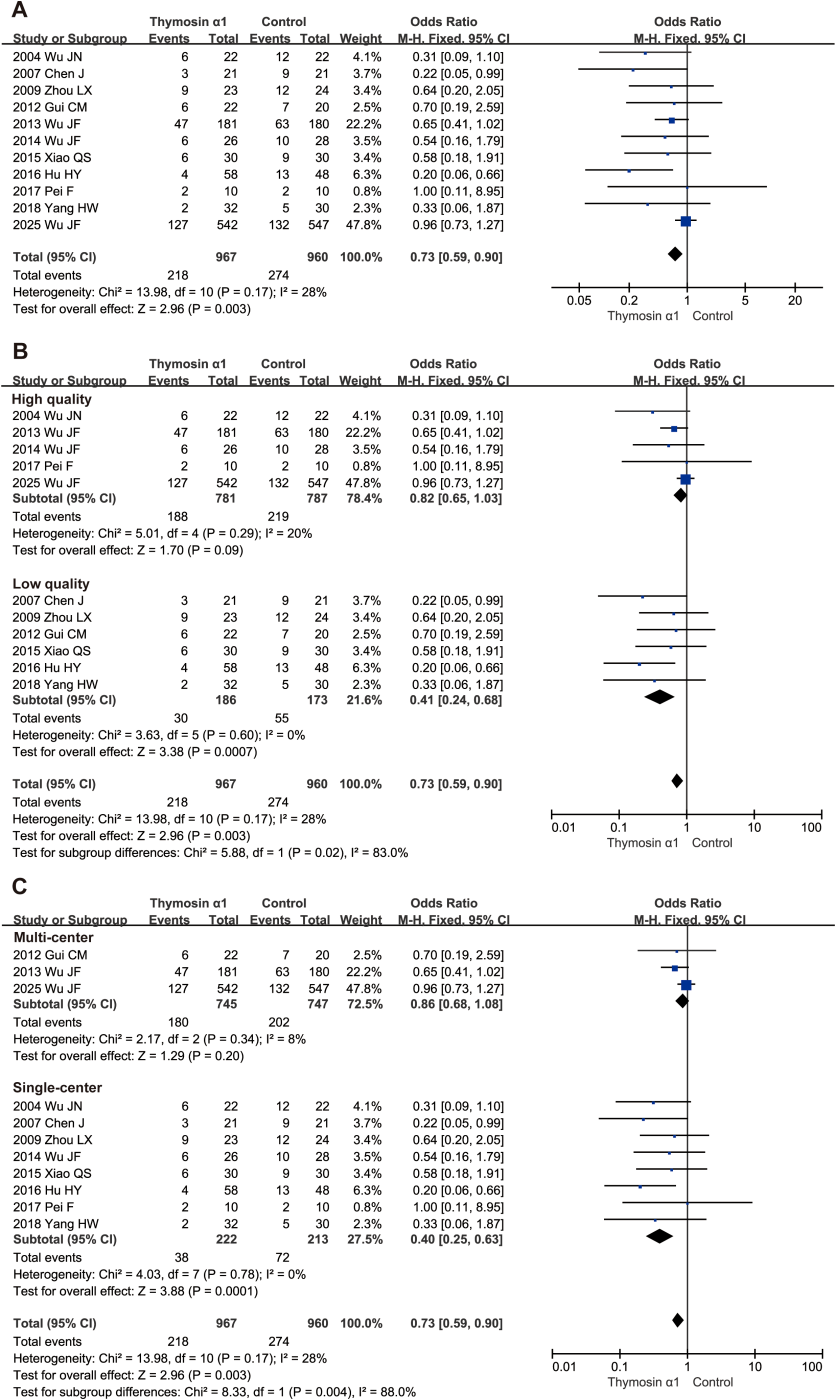
Forest plot of thymosin α1 on 28-day mortality. **(A)** pooled analysis of 11 RCTs, **(B)** subgroup analysis of relatively high- and low- quality studies, **(C)** subgroup analysis of multi-center and single-center studies.

A total of 1927 septic patients from 11 RCTs, including 967 in Tα1 group and 960 in control group, were evaluated for the 28-day mortality, with 218 and 274 deaths in the Tα1 and control groups, respectively. The results showed that Tα1 therapy significantly reduced 28-day mortality compared to controls (OR 0.73, 95%CI: 0.59-0.90, *P* = 0.003, **Figure 2A**). A meta-analysis including four RCTs without 28-day mortality data, showed similar results (**Supplementary Figure S2** **).**

Sensitivity analysis indicated this result was robust although one study had huge impact (**Supplementary Figure S3**). However, publication bias was evident as shown in the funnel plot (**Supplementary Figure S4**) and confirmed by Egger’s test (*P* = 0.01).

### Subgroup analysis for primary outcome

We conducted several subgroup analyses at the study level to further examine the primary outcome. First, we categorized studies by quality. Five higher-quality studies (1568 patients with sepsis) showed no significant benefit of Tα1 therapy (OR 0.82, 95% CI: 0.65–1.03, *P* = 0.09), while six lower-quality studies (359 patients) demonstrated a significant reduction in 28-day mortality (OR 0.41, 95% CI: 0.24–0.68, *P* = 0.0007, **Figure 2B**). Subgroup differences were significant (*P* = 0.02), with low heterogeneity in each subgroup. Second, we grouped studies by design into single-center and multi-center subgroups. Tα1 therapy significantly reduced 28-day mortality in single-center studies (OR 0.40, 95% CI: 0.25–0.63, *P* = 0.0001) but not in multi-center studies (OR 0.86, 95% CI: 0.68–1.08, *P* = 0.20, **Figure 2C**). Subgroup differences were significant (*P* = 0.004), with low heterogeneity in both subgroups. Third, regardless of whether the drug dosage was twice a day or once a day, the subgroup results both favored Tα1 treatment (OR 0.60, 95% CI: 0.37–0.96, *P* = 0.03 and OR 0.51, 95% CI: 0.29–0.91, *P* = 0.02 respectively, **Supplementary Figure S5**). In addition, two studies which followed sepsis 3.0 diagnosis criterion showed no significant 28-day survival benefit, while sum of the others suggested Tα1 treatment (**Supplementary Figure S6**).

**Figure 3:**
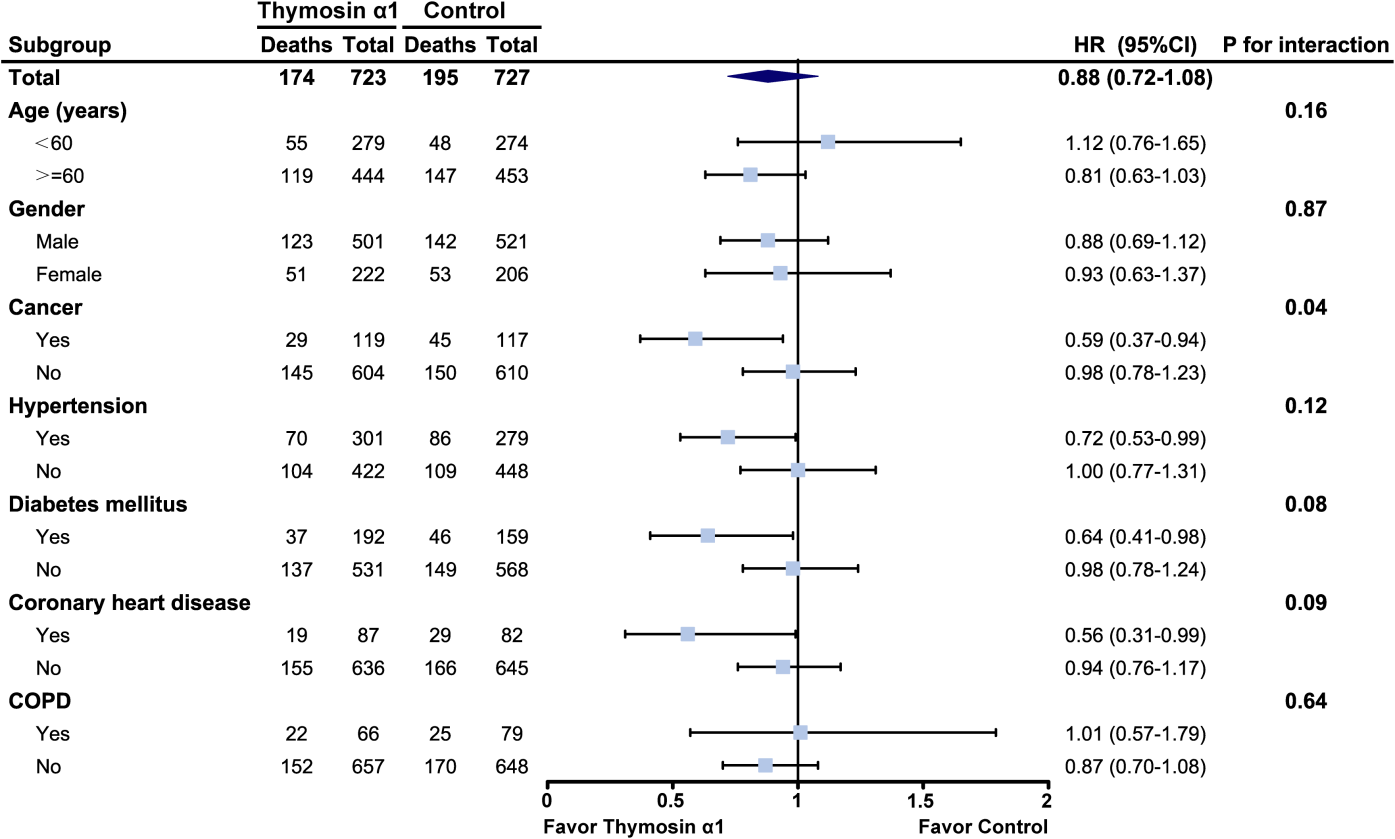
Heterogeneity of treatment effects analysis. Hazard ratio (HR) was adjusted by the studies. COPD, chronic obstructive pulmonary disease.

Then, we also conducted subgroup analyses at the patient level. The heterogeneity of treatment effects (HTE) was analyzed using individual data from two high-quality, multicenter RCTs (Wu et al., 2013; Wu et al., 2025), which including 75% patients of total. By day 28 post-randomization, 174 of 723 patients (24.1%) in the Tα1 group and 195 of 727 patients (26.8%) in the control group had died (HR 0.88, 95% CI: 0.72–1.08, **Figure 3**). HTE analysis across seven subgroups showed Tα1 improved 28-day survival in septic patients with cancer (HR 0.59, 95% CI: 0.37–0.94, *P*_interaction_ = 0.04; moderate credibility), diabetes (HR 0.64, 95% CI: 0.41–0.98; low credibility) and coronary heart disease (HR 0.56, 95% CI: 0.31–0.99; low credibility) (**Figure 3**). Detailed HTE analyses for three subgroups are provided in **Supplementary Material**-ICEMAN reports.

**Figure 4:**
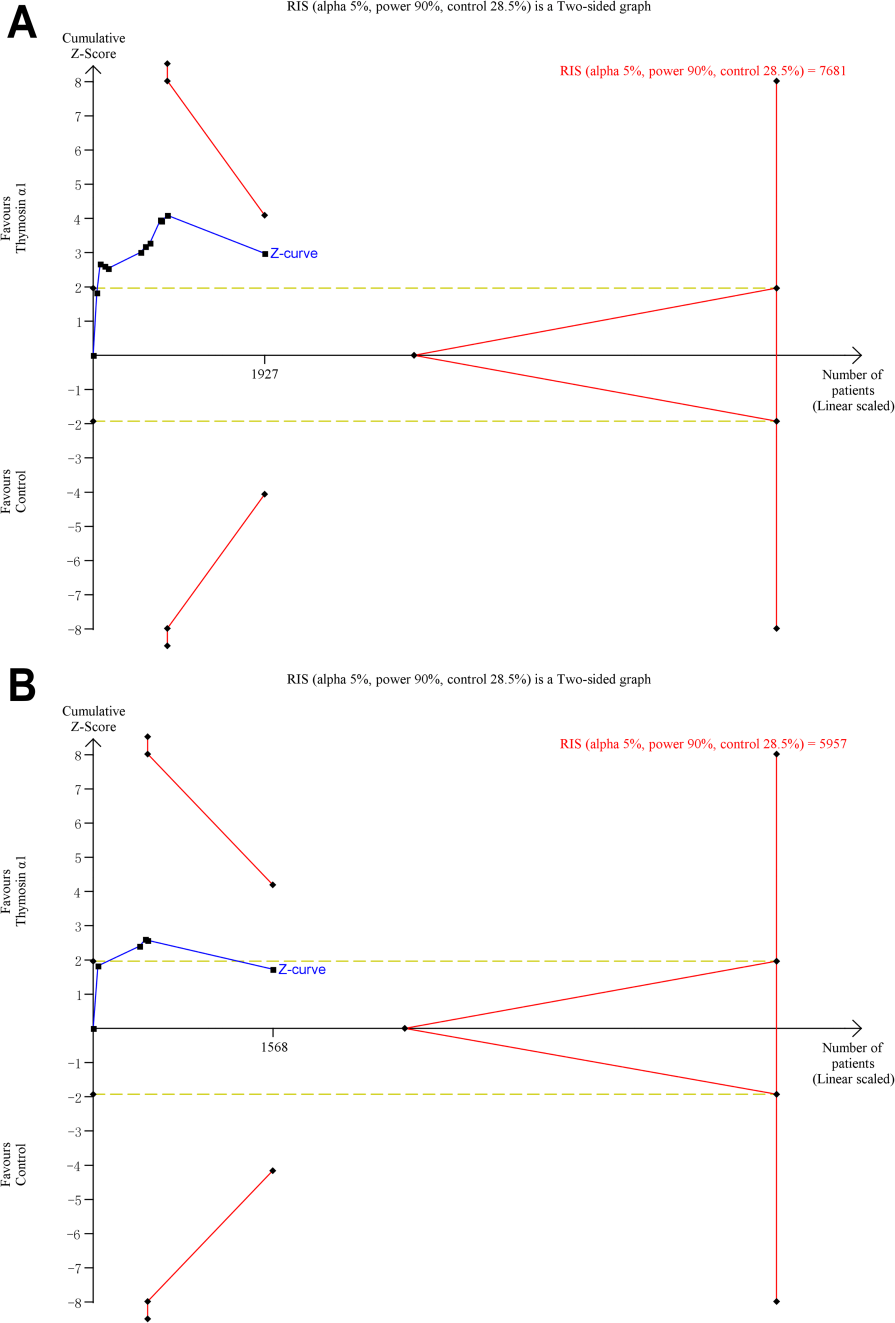
Trial sequential analysis for 28-day mortality. **(A)** all included 11 RCTs, **(B)** five relatively high-quality RCTs. The blue Z-curve represents the pooled odds ratio, with yellow dotted lines indicating conventional meta-analysis boundaries (5% alpha level). Trial sequential boundaries are shown by the symmetric red line above the Z-curve. Between the yellow dotted lines, the triangular futility zone indicates conclusive evidence that treatment effects fail to reach significance. RIS, required information size.

Furthermore, TSA graphs were presented in **Figure 4**, which revealed that the current systematic review did not achieve the required information size (RIS) to determine the effect on 28-day mortality. While the cumulative Z-curve from all 11 RCTs crossed conventional meta-analysis boundaries, it did not cross trial sequential boundaries, indicating a risk of false positives and the need for cautious interpretation. For the five higher-quality RCTs, the cumulative Z-curve did not cross conventional meta-analysis, trial sequential, or futility boundaries, underscoring the need for more rigorously designed studies to confirm the efficacy of Tα1 in patient with sepsis.

### The severity of sepsis

To assess the value of Tα1 in attenuating the disease severity in patients with sepsis, SOFA score and APACHE II score were compared. The pooled results indicated no significant difference in SOFA between Tα1 and control groups (mean difference: -0.38, 95%CI: -1.35 to 0.60, *P* = 0.45, **Supplementary Figure S5**). We found Tα1 therapy reduced APACHE II score more significantly compared to control group (mean difference: -2.81, 95%CI: -4.39 to -1.22, *P* = 0.0005, **Supplementary Figure S6**).

## Discussion

In this updated meta-analysis, we found that thymosin alpha 1 (Tα1) may reduce 28-day mortality in patients with sepsis compared to the control group. However, the reliability of the current evidence remains uncertain because the analysis did not reach the required sample size. Furthermore, both study-level and patient-level subgroup analyses exhibited high heterogeneity, indicating that future clinical trials should adopt personalized treatment strategies instead of a one-size-fits-all approach.

Tα1, as an immunomodulator, has the potential to improve prognosis by reestablishing immune homeostasis (Serafino et al., 2012; Pei et al., 2018; Aynekulu Mersha et al., 2025). In other acute diseases, its anti-inflammatory effects had been studied (Liu et al., 2025; Tian et al., 2025). Previous meta-analyses had suggested that Tα1, whether administrated as monotherapy or in conjunction with anti-inflammatory agents, could reduce mortality among septic patients (Li et al., 2015; Feng et al., 2016; Liu D. et al., 2016; Liu F. et al., 2016; Wang et al., 2016). Approximately a decade ago, Liu and colleagues (Liu F. et al., 2016) conducted an analysis of 10 randomized controlled trials encompassing 530 sepsis patients, proposing that Tα1 treatment might reduce mortality, although this conclusion was constrained by small sample sizes and low quality of evidence. Concurrently, a meta-analysis by Wang et al. (2016) investigated 944 sepsis patients across 6 randomized controlled trials, demonstrating that Tα1, when combined with ulinastatin, could improve short-term survival. Despite these early findings, a recent high-quality, multi-center randomized clinical trial failed to corroborate these results (Wu et al., 2025). This inconsistency prompted us to undertake an updated meta-analysis. Consistent with previous results, the findings of this study endorse the hypothesis that Tα1 reduces mortality in patients with sepsis. However, further TSA analysis reveals that the assertion regarding Tα1’s effect on reducing sepsis mortality remains inconclusive due to an inadequate sample size.

Sepsis presents as a highly heterogeneous clinical syndrome, wherein single therapeutic interventions often exhibit variable efficacy across different patient populations (Seymour et al., 2019; Shah et al., 2021; Pei et al., 2024). In this study, we assessed heterogeneity at two different levels: the study level and the patient level. At the study level, subgroups derived from multi-center studies and those with higher quality did not yield results consistent with the overall findings. This underscores the critical importance of conducting high-quality, multi-center studies in sepsis immunology research. At the patient level, multiple subgroups with chronic diseases also exhibited significant treatment heterogeneity. Although these findings considerably undermine our primary results, it may also indicate potential target populations for Tα1 treatment. As Kalil et al. (2025) stated, stratifying sepsis patients into subphenotypes may aid in elucidating the complexities of sepsis.

Interestingly, our subgroup analysis suggests that patients with chronic conditions may exhibit heightened responsiveness to Tα1 therapy. This observation is in consistent with the immunomodulatory effects of the drug observed in elderly COVID-19 patients (Liu et al., 2020; Yu et al., 2020). Recent studies have showed that Tα1 therapy can benefit elderly COVID-19 patients by modulating T lymphocyte responses, specifically by increasing CD4^+^ and CD8^+^ T cell populations while preventing excessive activation of CD8^+^ T cells (Liu et al., 2020; Yu et al., 2020). The increasing prevalence of chronic diseases introduces additional complexity to sepsis treatment (Zheng et al., 2018; Zhou et al., 2021). These conditions can cause sustained damage to the immune system and may exacerbate the already compromised immune function in sepsis patients, potentially leading to immunosuppression (Mian et al., 2014; Pene et al., 2016; Frydrych et al., 2017; Trevelin et al., 2017; Mikolajczyk and Guzik, 2019). Although our findings suggest that patients with chronic conditions might be an appropriate target population for Tα1 treatment, this hypothesis requires validation through high-quality clinical trials before definitive conclusions can be established.

While we have compared different administration methods of Tα1, the optimal dosage for sepsis treatment remains undetermined. Lin (2007) identified a dose-dependent effect of Tα1, with administration twice daily resulting a prognostic improvement. A subgroup analysis within this study revealed that both once-daily and twice-daily dosing reduced 28-day mortality, suggesting that twice-daily administration was safe. In addition, similarly to the therapeutic dose, there is insufficient evidence regarding the optimal therapeutic duration of Tα1. Current clinical studies have set the therapeutic course at seven days, which may facilitate study implementation. However, the restoration of immune function in sepsis patients is a prolonged process, with some patients experiencing immune imbalance for three weeks or longer (Inoue et al., 2013; Stortz et al., 2018). Consequently, real-world studies are necessary to further ascertain the appropriate therapeutic duration.

### Strengths and limitations

The present study has several notable strengths. First, our comprehensive systematic search and rigorous quality assessment, alongside the exclusive inclusion of RCTs, have enhanced the reliability of our findings. Second, we have innovatively applied TSA to evaluate the efficacy of Tα1, thereby providing valuable insights for future trial design. Third, our heterogeneity analyses, which utilizes individual patient data from two high-quality multicenter RCTs (accounting for 75% of the total study population) and is validated by ICEMAN, offers a rigorous evaluation of potential effect modifications. These methodological strengths collectively render our study the most rigorous evaluation of Tα1 treatment in sepsis to date.

However, this meta-analysis also has several limitations that should be acknowledged. First, all included studies were conducted in China, raising concerns about the generalizability of the findings to other racial or ethnic populations. Second, the potential for publication bias cannot be ruled out, particularly due to the influence of the recent TESTS study (Wu et al., 2025). Third, a significant limitation of the included studies lies in their small sample sizes. Ten out of fourteen studies enrolled fewer than 100 participants, potentially diminishing the statistical power and reliability of the results. Fourth, this analysis exclusively considered 28-day mortality as the primary outcome measure, due to incomplete or inconsistently reported data on ICU and in-hospital across many studies. We strongly recommended that future randomized controlled trials implement standardized and comprehensive outcome reporting. Fifth, to specifically assess the effect of Tα1, combination therapies (e.g., ulinastatin or continuous renal replacement therapy) were excluded, which may limit the applicability of our findings to real-world clinical settings where such combination therapies are prevalent. Sixth, the diagnostic criteria for sepsis varied across the included studies. Over the past two decades, sepsis definitions have been undergone multiple refinements, leading to inconsistencies in diagnosis and treatment strategies, which may contribute to heterogeneity in the results.

## Conclusion

Recent evidence suggests that Tα1 may reduce 28-day mortality in patients with sepsis. However, it is important to emphasize that the current sample size is insufficient to confirm this conclusion. Furthermore, the efficacy of Tα1 varies significantly among different subgroups, suggesting that uniform clinical trials across the entire sepsis population may not be appropriate. Future research in immunotherapy should focus on developing personalized treatment strategies to improve therapeutic efficacy and patient outcomes.

## Data Availability

The original contributions presented in the study are included in the article/**Supplementary Material**. Further inquiries can be directed to the corresponding author.
