# Supplementary material for: Efficacy of thymosin α1 for sepsis: a systematic review and meta-analysis of randomized controlled trials
Source: Front Cell Infect Microbiol. 2025 Sep 3;15:1673959. doi: 10.3389/fcimb.2025.1673959 (PMC12440967; doi:10.3389/fcimb.2025.1673959)
Supplement: Supplementary file 1 [file Table1.docx]

**Supplementary Material**

**Contents**

[**1. Search strategy 2**](#_Toc203582222)

[**2. Supplementary figures and tables 3**](#_Toc203582223)

[**Figure S1. Quality assessment 3**](#_Toc203582224)

[**Figure S2. Forest plot of thymosin α1 on undefined mortality 4**](#_Toc203582225)

[**Figure S3. Sensitivity analysis 5**](#_Toc203582226)

[**Figure S4. Funnel plot to assess publication bias 6**](#_Toc203582227)

[**Figure S5. Forest plot of subgroup analysis by doses of thymosin α1. 7**](#_Toc203582228)

[**Figure S6. Forest plot of subgroup analysis by applied Surviving Sepsis Campaign guidelines. 8**](#_Toc203582229)

[**Figure S7. Forest plot of thymosin α1 on SOFA score 9**](#_Toc203582230)

[**Figure S8. Forest plot of thymosin α1 on APACHE II score 9**](#_Toc203582231)

[**Table S1. Additional characteristics of the included studies 10**](#_Toc203582232)

[**3. ICEMAN reports 13**](#_Toc203582233)

[**(1) Credibility assessment for cancer subgroup 13**](#_Toc203582234)

[**(2) Credibility assessment for coronary heart disease subgroup 15**](#_Toc203582235)

[**(3) Credibility assessment for diabetes subgroup 17**](#_Toc203582236)

[**4. PRISMA 2020 checklist 19**](#_Toc203582237)

1. Search strategy

**(1). PubMed**

**Search**: (thymosin OR thymosin alpha1 OR thymus OR maipuxin OR thymalfasin OR zadaxin) AND sepsis

**Advanced**：

("thymosin"[MeSH Terms] OR "thymosin"[All Fields] OR "thymosins"[All Fields] OR "thymosine"[All Fields] OR ("thymalfasin"[MeSH Terms] OR "thymalfasin"[All Fields] OR ("thymosin"[All Fields] AND "alpha1"[All Fields]) OR "thymosin alpha1"[All Fields]) OR ("thymus plant"[MeSH Terms] OR ("thymus"[All Fields] AND "plant"[All Fields]) OR "thymus plant"[All Fields] OR "thymus"[All Fields] OR "thymus gland"[MeSH Terms] OR ("thymus"[All Fields] AND "gland"[All Fields]) OR "thymus gland"[All Fields] OR "thymus"[All Fields] OR "thymus extracts"[MeSH Terms] OR ("thymus"[All Fields] AND "extracts"[All Fields]) OR "thymus extracts"[All Fields]) OR ("maipuxin"[Supplementary Concept] OR "maipuxin"[All Fields]) OR ("thymalfasin"[MeSH Terms] OR "thymalfasin"[All Fields]) OR ("thymalfasin"[MeSH Terms] OR "thymalfasin"[All Fields] OR "zadaxin"[All Fields])) AND ("sepsis"[MeSH Terms] OR "sepsis"[All Fields])

**(2). Web of Science**

**Search**: (thymosin OR thymosin alpha1 OR thymus OR maipuxin OR thymalfasin OR zadaxin) AND sepsis (Topic)

**(3). Embase**

**Search**: ('thymosin'/exp OR thymosin OR 'thymosin alpha1'/exp OR 'thymosin alpha1' OR (('thymosin'/exp OR thymosin) AND alpha1) OR 'thymus'/exp OR thymus OR maipuxin OR 'thymalfasin'/exp OR thymalfasin OR 'zadaxin'/exp OR zadaxin) AND ('sepsis'/exp OR sepsis)

**(4). Cochrane library**

**Search**: (thymosin OR thymosin alpha1 OR thymus OR maipuxin OR thymalfasin OR zadaxin) AND sepsis

**(5). China National Knowledge Internet (CNKI)**

**Search**：篇关摘：胸腺肽 脓毒症

(Translation) Title, key word and abstract: thymosin and sepsis

**(6). Wanfang data**

**Search**：全部：胸腺肽 脓毒症

(Translation) All: thymosin and sepsis

**(7). China Science and Technology Journal Database (VIP)**

**Search**: 标题或关键词: 胸腺肽 脓毒症

(Translation) Title or key word: thymosin and sepsis

1. Supplementary figures and tables


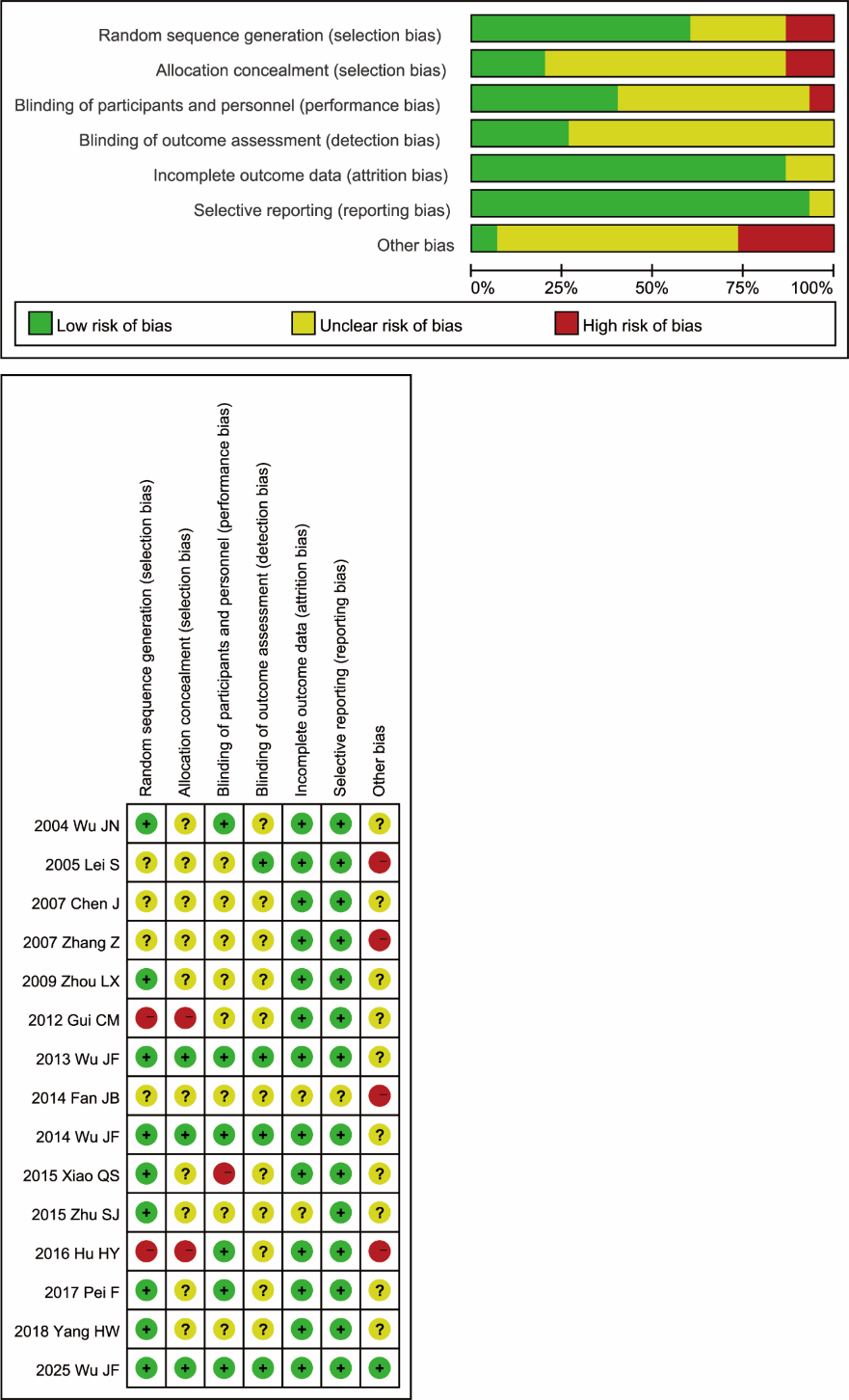
**Figure S1**. Quality assessment

15 RCTs were assessed by the Cochrane Collaboration’s tool for assessing risk of bias. Red dots represent high risk of bias. Yellow dots represent unclear risk of bias. Green dots represent low risk of bias.

**Figure S2. Forest plot of thymosin α1 on undefined mortality**


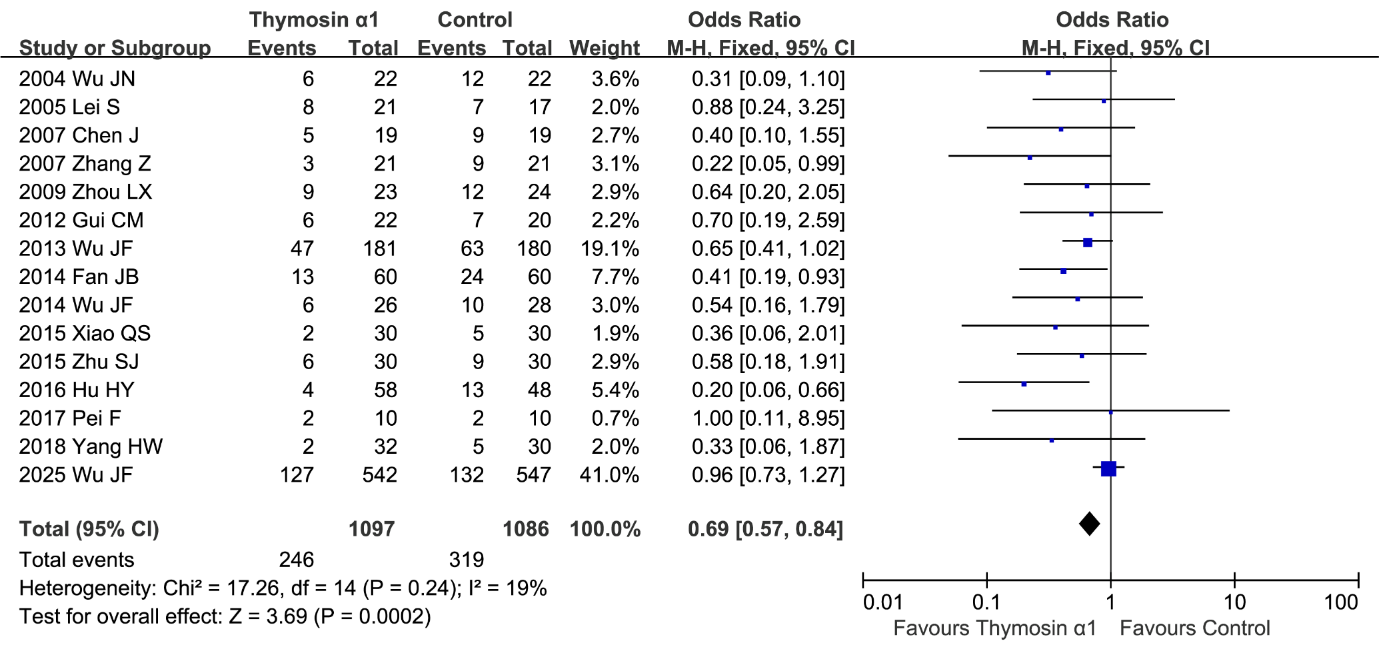


15 RCTs were synthesized using fixed model and Mantel-Haenszel test. Undefined mortality included ICU mortality, hospital mortality and 28-day mortality.

**Figure S3**. Sensitivity analysis


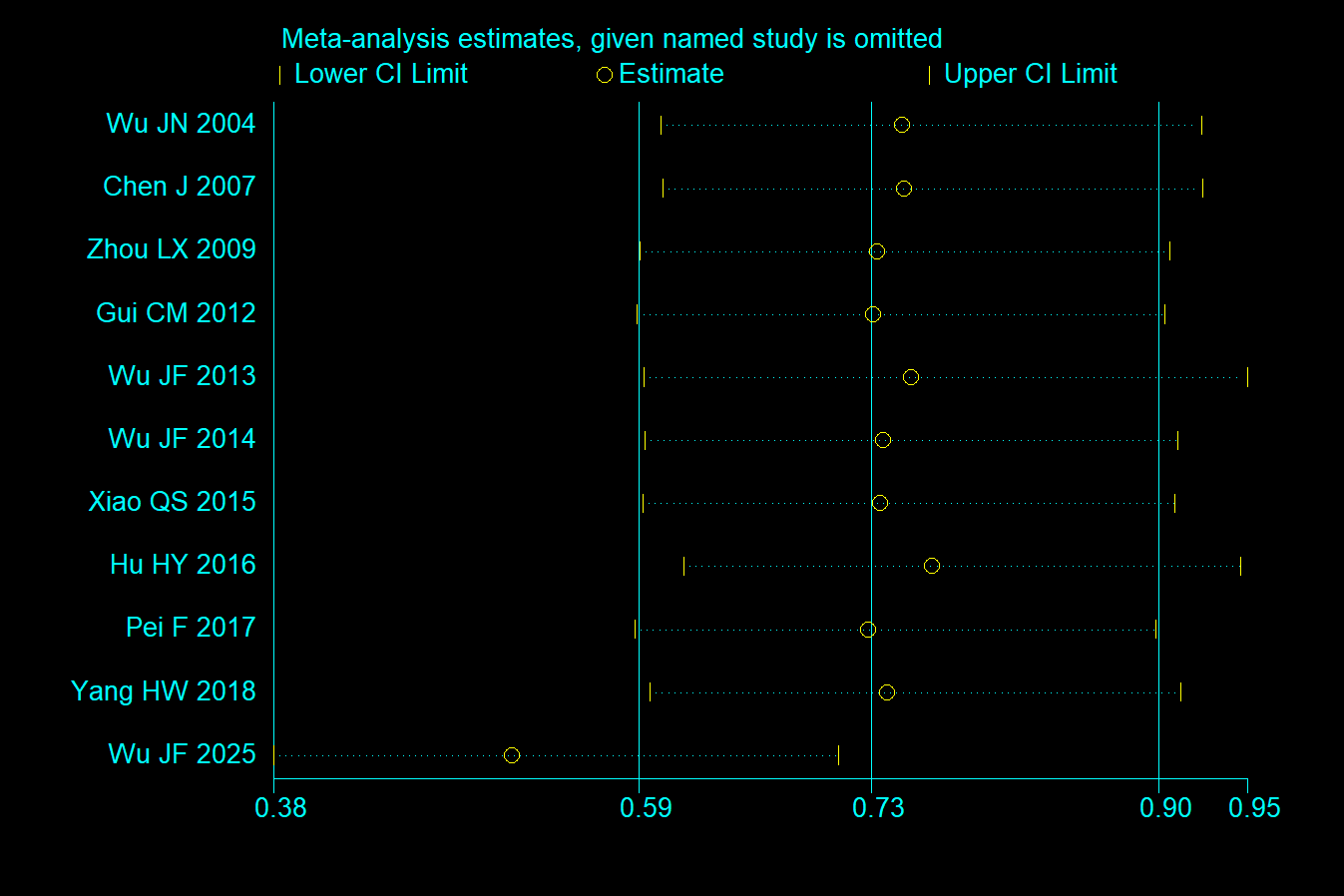


Sensitivity analysis of 28-day mortality. Odds ratio was reported through fixed model and Mantel-Haenszel test.

**Figure S4**. Funnel plot to assess publication bias


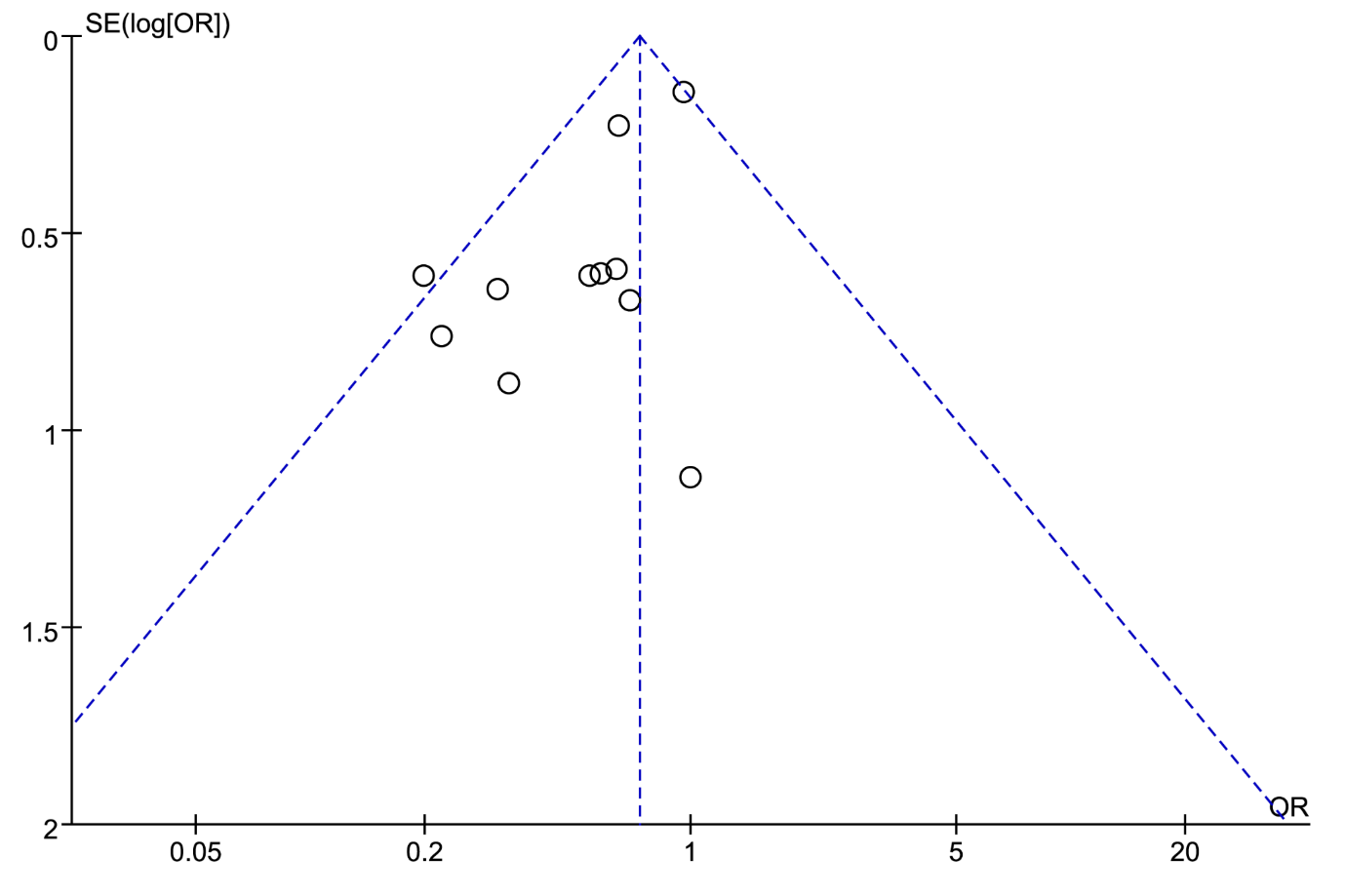


Each circle represents a study. OR: odds ratio. SE: standard error.

**Figure S5.** Forest plot of subgroup analysis by doses of **thymosin α1.**


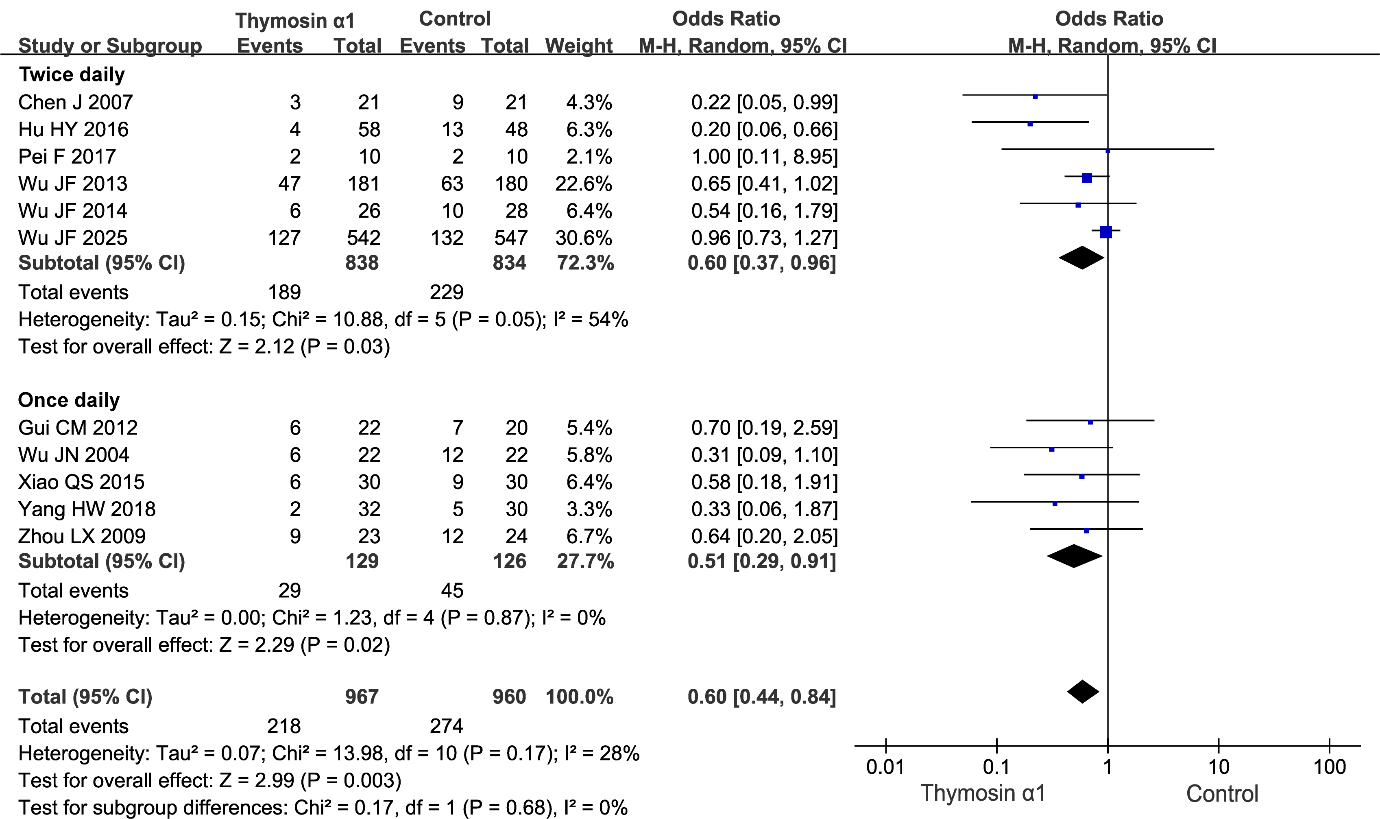


**Figure S6.** Forest plot of subgroup analysis by applied Surviving Sepsis Campaign guidelines**.**


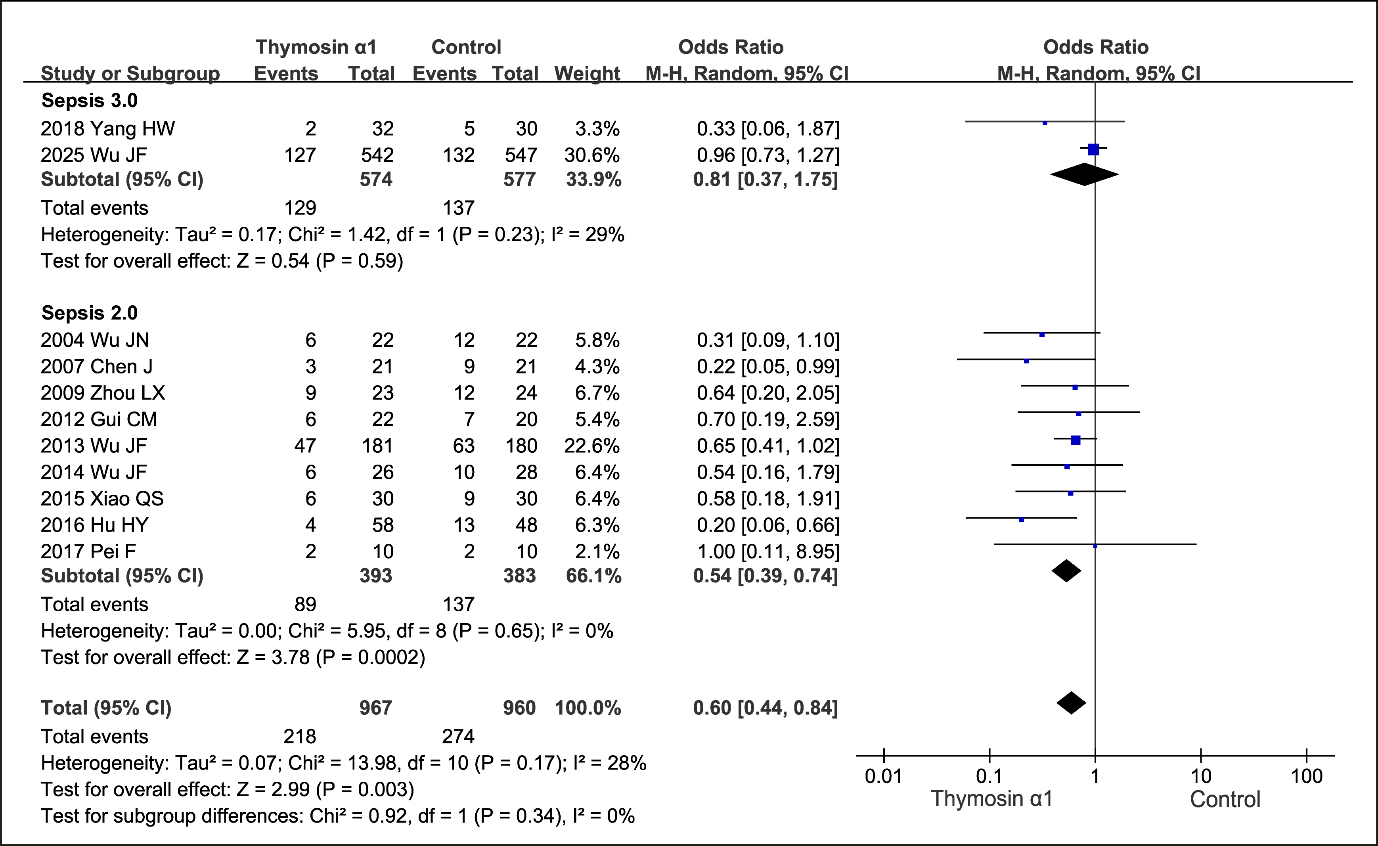


**Figure S7**. Forest plot of **thymosin α1 on** SOFA score


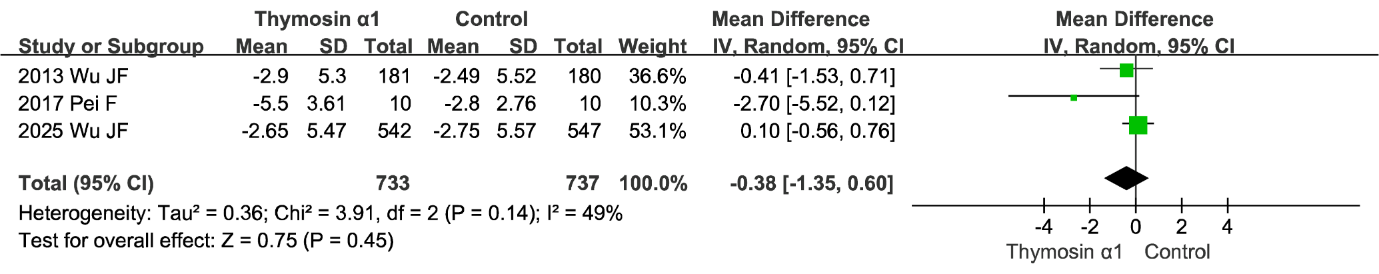


Mean difference was reported through random model.

**Figure S8**. Forest plot of **thymosin α1 on** APACHE II score


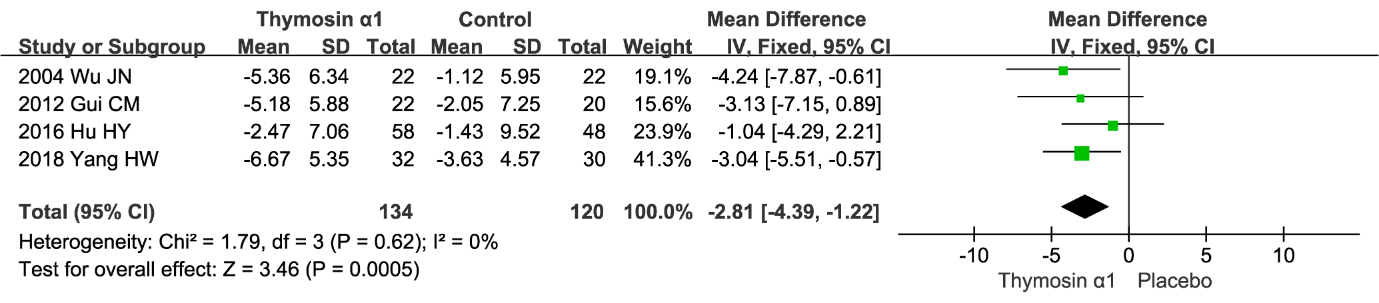


Mean difference was reported through fixed model.

**Table S1**. Additional characteristics of the included studies

| **Study** | **Intervention (thymosin α1) group** | | | | **Control group** | | | |
| --- | --- | --- | --- | --- | --- | --- | --- | --- |
|  | **Deaths/Total** | **Age** | **Gender**  **(male/female)** | **Treatment** | **Deaths/Total** | **Age** | **Gender**  **(male/female)** | **Treatment** |
| 2004 Wu JN et al. | 6/22 | N/A | N/A | 1.6mg every day for ten days | 12/22 | N/A | N/A | Conventional treatment |
| 2005 Lei S et al. | 8/21 | 68±20 | 14/7 | 1.6mg twice a day for one week | 7/17 | 66±16 | 11/6 | Conventional treatment |
| 2007 Zhang Z et al. | 5/19 | N/A | N/A | 1.6mg every day for at least five days | 9/19 | N/A | N/A | Conventional treatment |
| 2007 Chen J et al. | 3/21 | 42 (19-75) | 13/8 | 1.6mg twice a day for one week | 9/21 | 45 (22-78) | 14/7 | 2004 International guidelines for septic shock |
| 2009 Zhou LX et al. | 9/23 | 57±19 | 14/9 | 1.6mg every day for one week | 12/24 | 60±20 | 15/9 | surviving sepsis campaign: guidelines for sepsis and septic shock |
| 2012 Gui CM et al. | 6/22 | 52±15 | 16/6 | 1.6mg every day for one week | 7/20 | 50±13 | 15/5 | 2008 surviving sepsis campaign: guidelines for sepsis and septic shock |
| 2013 Wu JF et al. | 47/181 | 65±15 | 141/40 | 1.6mg twice a day for five days and then every for two days | 63/180 | 66±13 | 131/49 | 2008 surviving sepsis campaign: guidelines for sepsis and septic shock |
| 2014 Wu JF et al. | 6/26 | 60±12 | 21/5 | 1.6mg twice a day for five days and then every for two days | 10/28 | 61±11 | 20/8 | 2012 surviving sepsis campaign: guidelines for sepsis and septic shock |
| 2014 Fan JB et al. | 13/60 | N/A | N/A | 1.6mg every day for one week | 24/60 | N/A | N/A | 2004 International guidelines for septic shock |
| 2015 Zhu SJ et al. | 2/30 | 44±13 | 15/15 | 1.6mg every three days for two weeks | 5/30 | 41±11 | 16/14 | 2004 International guidelines for septic shock |
| 2015 Xiao QS et al. | 6/30 | 66±11 | 15/15 | 1.6mg every day for six days | 9/30 | 65±10 | 16/14 | surviving sepsis campaign: guidelines for sepsis and septic shock |
| 2016 Hu HY et al. | 4/58 | 42±11 | N/A | 1.6mg twice a day for five days | 13/48 | 41±11 | N/A | 2012 surviving sepsis campaign: guidelines for sepsis and septic shock |
| 2016 Wang W et al. | 54/127 | 63±16 | 83/44 | 1.6mg twice a day for one week | 59/117 | 63±17 | 86/31 | 2008 surviving sepsis campaign: guidelines for sepsis and septic shock |
| 2017 Pei F et al. | 2/10 | 61±12 | 7/3 | 1.6mg twice a day for one week | 2/10 | 57±16 | 3/7 | 2012 surviving sepsis campaign: guidelines for sepsis and septic shock; saline as placebo |
| 2017 Ouyang Q et al. | 14/39 | N/A | N/A | 1.6mg every day for one week | 15/32 | N/A | N/A | Conventional treatment |
| 2018 Yang HW et al. | 2/32 | 60±19 | 22/10 | 1.6mg every day for one week | 5/30 | 60±20 | 20/10 | Conventional treatment |
| 2018 Su HY et al. | 8/34 | 86±5 | 31/3 | 1.6mg every three days for two weeks | 12/34 | 83±10 | 27/7 | Conventional treatment |
| 2025 Wu JF et al. | 127/542 | 65 (52-74) | 360/182 | 1.6mg every 12 hours for one week | 132/547 | 65 (51-72) | 390/157 | 2016 surviving sepsis campaign: guidelines for sepsis and septic shock |

(continued)

| **Study** | **Length of stay in ICU (days)** | | **Mechanical ventilation (days)** | | **SOFA score^a^** | | **APACHE II score^a^** | | **Study quality score** |
| --- | --- | --- | --- | --- | --- | --- | --- | --- | --- |
|  | **intervention** | **control** | **intervention** | **control** | **intervention** | **control** | **intervention** | **control** |  |
| 2004 Wu JN et al. | N/A | N/A | N/A | N/A | N/A | N/A | 15.6±4.5 | 19.1±4.5 | 11 |
| 2005 Lei S et al. | 19±6 | 27±10 | 15±7 | 2±10 | N/A | N/A | 25.6±5.7 | 24.0±4.1 | 9 |
| 2007 Zhang Z et al. | 15±4 | 18±5 | 9±3 | 13±4 | N/A | N/A | 18.4±3.8 | 18.1±3.6 | 8 |
| 2007 Chen J et al. | 9±4 | 13±3 | 5±4 | 8±4 | N/A | N/A | N/A | N/A | 9 |
| 2009 Zhou LX et al. | 15±9 | 16±9 | 11±5 | 12±6 | N/A | N/A | N/A | N/A | 10 |
| 2012 Gui CM et al. | N/A | N/A | N/A | N/A | N/A | N/A | 8.7±3.6 | 12.6±5.8 | 7 |
| 2013 Wu JF et al. | 11 (7-20) | 11 (5-21) | 7 (3-13) | 6 (2-14) | 5.3 (4.5-6.2) | 5.9 (5.0-6.7) | N/A | N/A | 13 |
| 2014 Wu JF et al. | N/A | N/A | N/A | N/A | N/A | N/A | N/A | N/A | 13 |
| 2014 Fan JB et al. | N/A | N/A | N/A | N/A | N/A | N/A | 33.6±9.4 | 32.8±8.9 | 6 |
| 2015 Zhu SJ et al. | N/A | N/A | N/A | N/A | N/A | N/A | 15.0±2.8 | 14.5±2.7 | 9 |
| 2015 Xiao QS et al. | 6±2 | 8±2 | N/A | N/A | N/A | N/A | N/A | N/A | 9 |
| 2016 Hu HY et al. | N/A | N/A | N/A | N/A | N/A | N/A | 15.1±6.3 | 18.0±7.8 | 7 |
| 2016 Wang W et al. | N/A | N/A | N/A | N/A | 7.0 (4.0-10.8) | 7.0 (5.0-9.0) | 20.5 (16.0-26.0) | 21.0 (15.0-27.0) | N/A |
| 2017 Pei F et al. | 6±3 | 6±2 | 4±3 | 4±3 | 4.6±2.5 | 6.8±1.9 | N/A | N/A | 11 |
| 2017 Ouyang Q et al. | 15±3 | 18±4 | 12±4 | 15±4 | 3.8±1.5 | 7.4±1.9 | 10.5±3.4 | 13.7±4.8 | N/A |
| 2018 Yang HW et al. | 15±3 | 20±6 | N/A | N/A | N/A | N/A | 15.7±4.2 | 18.3±2.9 | 10 |
| 2018 Su HY et al. | N/A | N/A | N/A | N/A | N/A | N/A | N/A | N/A | N/A |
| 2025 Wu JF et al. | 15 (8-28) | 15 (8-28) | N/A | N/A | 4.9±4.0 | 4.8±4.0 | N/A | N/A | 14 |

^a^ data on day 7 from ICU admission.

Three retrospective studies were not assessed for risk of bias.

N/A, not applicable.

1. ICEMAN reports
2. **Credibility assessment for cancer subgroup**

| **CREDIBILITY ASSESSMENT** | | | | |
| --- | --- | --- | --- | --- |
| **Essential preliminary considerations to define the possible effect modification of interest** | | | |  |
| State a single candidate effect modifier (e.g., age or comorbidity): Cancer | | | |  |
| Was the effect modifier measured before or at randomization? [**x**] yes, continue [ ] no, stop here and refer to manual for further instructions | | | |  |
| State a single outcome and time-point (e.g., mortality at 1 year follow-up): Mortality at 28-day follow-up | | | |  |
| State a single effect measure (e.g., relative risk or risk difference): Hazard ratio | | | |  |
| **1: Is the analysis of effect modification based on comparison within rather than between trials?** | | | | |
| [ ] Completely between | [ ] Mostly between or unclear | [ ] Mostly within | [**x**] Completely within | |
| *Subgroup analysis or meta-regression comparing overall effects of each individual trial. This is typical for aggregate data meta-analysis.* | *Subgroup analysis or meta-regression with most information coming from overall effects, but some trials providing within-trial subgroup information* | *Most trials providing within-trial subgroup information; or individual participant data analysis that combines within and between trial information* | *All trials providing within-trial subgroup information or individual participant data; and the analysis separates within from between trial information, e.g., meta-analysis of interactions* | |
| Comment: We acquired within-trial subgroup information of ETASS and TESTS, the patients were summarized to study interactions. | | | | |
| **2: For within-trial comparisons, is the effect modification similar from trial to trial?** [ ] Not applicable: no or one within-RCT comparison | | | | |
| [ ] Definitely not similar | [ ] Probably not similar or unclear | [**x**] Mostly similar | [ ] Definitely similar | |
| *Effect modification reported for two or more trials and clearly different directions* | *Effect modification not reported for individual trials or too imprecise to tell* | *Effect modification reported for two or more trials, mostly similar in direction, but considerable differences in magnitude* | *Effect modification reported for two or more trials, similar in direction, only some differences in magnitude* | |
| Comment: The direction of effect modification was mostly similar between two trials. | | | | |
| **3: For between-trial comparisons, is the number of trials large?** [ ] Not applicable: no between RCT comparison | | | | |
| [**x**] Very small | [ ] Rather small or unclear | [ ] Rather large | [ ] Large | |
| *1 or 2 or in smallest subgroup; 5 or less in continuous meta-regression* | *3-4 in smallest subgroup; 6-10 in continuous meta-regression* | *5-9 in smallest subgroup; 11 to 15 in continuous meta-regression* | *10 or more in smallest subgroup; more than 15 in continuous meta-regression* | |
| Comment: Two trials compared. | | | | |
| **4: Was the direction of effect modification correctly hypothesized a priori?** | | | | |
| [ ] Definitely no | [**x**] Probably no or unclear | [ ] Probably yes | [ ] Definitely yes | |
| *Clearly post-hoc or results inconsistent with hypothesized direction or biologically very implausible* | *Vague hypothesis or hypothesized direction unclear* | *No prior protocol available but unequivocal statement of a priori hypothesis with correct direction of effect modification* | *Prior protocol available and includes correct specification of direction of effect modification, e.g., based on a biologic rationale* | |
| Comment: Authors did not hypothesize clear direction of each specific effect modification, only hypothesized overall direction. | | | | |
| **5: Does a test for interaction suggest that chance is an unlikely explanation of the apparent effect modification?** (consider irrespective of number of effect modifiers) | | | | |
| [ ] Chance a very likely explanation | [**x**] Chance a likely explanation or unclear | [ ] Chance may not explain | [ ] Chance an unlikely explanation | |
| *Interaction or meta-regression p-value >0.05* | *Interaction or meta-regression p-value ≤0.05 and >0.01, or no test of interaction reported and not computable* | *Interaction or meta-regression p-value ≤0.01 and >0.005* | *Interaction or meta-regression p-value ≤0.005* | |
| Comment: Subgroup of whether cancer or not had interaction p-value of 0.04. | | | | |
| **6: Did the authors test only a small number of effect modifiers or consider the number in their statistical analysis?** | | | | |
| [ ] Definitely no | [**x**] Probably no or unclear | [ ] Probably yes | [ ] Definitely yes | |
| *Explicitly exploratory analysis or large number of effect modifiers tested (e.g., greater than 10) and multiplicity not considered in analysis* | *No mention of number or 4-10 effect modifiers tested and number not considered in analysis* | *No protocol available but unequivocal statement of 3 or fewer effect modifiers tested* | *Protocol available and 3 or fewer effect modifiers tested or number considered in analysis* | |
| Comment: ETASS trial had seven effect modifiers and TESTS trial had eight effect modifiers. | | | | |
| **7: Did the authors use a random effects model?** | | | | |
| [ ] Definitely no | [ ] Probably no or unclear | [**x**] Probably yes | [ ] Definitely yes | |
| *Fixed (or common) effect or fixed effects model explicitly stated* | *Probably fixed effect(s) model* | *Probably random (or mixed) effects* | *Random (or mixed) effects explicitly stated* | |
| Comment: A model with random intercepts was used. | | | | |
| **8: If the effect modifier is a continuous variable, were arbitrary cut points avoided?** [**x**] not applicable: not continuous | | | | |
| [ ] Definitely no | [ ] Probably no or unclear | [ ] Probably yes | [ ] Definitely yes | |
| *Analysis based on exploratory cut point(s), e.g., picking cut point associated with highest interaction p-value* | *Analysis based on cut point(s) of unclear origin* | *Analysis based on pre-specified cut point(s), e.g., suggested by prior RCT* | *Analysis based on the full continuum, e.g., assuming a linear or logarithmic relationship* | |
| Comment: Caner is a dichotomous variable. | | | | |
| **9 Optional: Are there any additional considerations that may increase or decrease credibility?** (manual section 3.9) [ ] not applicable | | | | |
|  | [ ] Yes, probably decrease | [**x**] Yes, probably increase | | |
| Comment: The two trials exhibited a low risk of bias, and thymosin α1 showed potential to enhance immune dysfunction in chronic conditions.   \| **10: How would you rate the overall credibility of the proposed effect modification?**  The overall rating should be driven by the items that decrease credibility. The following provides a sensible strategy:   - All responses definitely or probably decrease credibility or unclear 🡪 very low - Two or more responses definitely decrease credibility 🡪 maximum usually low even if all other responses satisfy credibility criteria - One response definitely decreases credibility 🡪 maximum usually moderate even if all other responses satisfy credibility criteria - Two responses probably decrease credibility 🡪 maximum usually moderate even if all other responses satisfy credibility criteria - No response options definitely or probably decrease credibility 🡪 high very likely   Place a mark on the continuous line (or type “x” in editable version) \| \| \| \| \|  \| \| --- \| --- \| --- \| --- \| --- \| --- \| \|  \|  \| \| \| \|  \| \|  \|  \| \| \| \|  \| \|  \|  \| \|  \|  \| \| \| \|  \| \|  \|  \| \| \| \|  \| \|  \| **Very low credibility** \| **Low credibility** \| **Moderate credibility** \| **High credibility** \|  \| \|  \|  \|  \|  \|  \|  \| \|  \| Minimal to no support for effect modification;  Use overall effect for each subgroup \| Some but insufficient support for effect modification;  Use overall effect for each subgroup but note remaining uncertainty \| Likely effect modification;  Use separate effects for each subgroup but note remaining uncertainty \| Very likely effect modification;  Use separate effects for each subgroup \|  \| \| Comment: Cancer is a likely effect modification, there remains uncertainty. \| \| \| \| \| \| | | | | |

1. **Credibility assessment for coronary heart disease subgroup**

| **CREDIBILITY ASSESSMENT** | | | | |
| --- | --- | --- | --- | --- |
| **Essential preliminary considerations to define the possible effect modification of interest** | | | |  |
| State a single candidate effect modifier (e.g., age or comorbidity): Coronary heart disease | | | |  |
| Was the effect modifier measured before or at randomization? [**x**] yes, continue [ ] no, stop here and refer to manual for further instructions | | | |  |
| State a single outcome and time-point (e.g., mortality at 1 year follow-up): Mortality at 28-day follow-up | | | |  |
| State a single effect measure (e.g., relative risk or risk difference): Hazard ratio | | | |  |
| **1: Is the analysis of effect modification based on comparison within rather than between trials?** | | | | |
| [ ] Completely between | [ ] Mostly between or unclear | [ ] Mostly within | [**x**] Completely within | |
| *Subgroup analysis or meta-regression comparing overall effects of each individual trial. This is typical for aggregate data meta-analysis.* | *Subgroup analysis or meta-regression with most information coming from overall effects, but some trials providing within-trial subgroup information* | *Most trials providing within-trial subgroup information; or individual participant data analysis that combines within and between trial information* | *All trials providing within-trial subgroup information or individual participant data; and the analysis separates within from between trial information, e.g., meta-analysis of interactions* | |
| Comment: We acquired within-trial subgroup information of ETASS and TESTS, the patients were summarized to study interactions. | | | | |
| **2: For within-trial comparisons, is the effect modification similar from trial to trial?** [ ] Not applicable: no or one within-RCT comparison | | | | |
| [ ] Definitely not similar | [ ] Probably not similar or unclear | [**x**] Mostly similar | [ ] Definitely similar | |
| *Effect modification reported for two or more trials and clearly different directions* | *Effect modification not reported for individual trials or too imprecise to tell* | *Effect modification reported for two or more trials, mostly similar in direction, but considerable differences in magnitude* | *Effect modification reported for two or more trials, similar in direction, only some differences in magnitude* | |
| Comment: The direction of effect modification was mostly similar between two trials. | | | | |
| **3: For between-trial comparisons, is the number of trials large?** [ ] Not applicable: no between RCT comparison | | | | |
| [**x**] Very small | [ ] Rather small or unclear | [ ] Rather large | [ ] Large | |
| *1 or 2 or in smallest subgroup; 5 or less in continuous meta-regression* | *3-4 in smallest subgroup; 6-10 in continuous meta-regression* | *5-9 in smallest subgroup; 11 to 15 in continuous meta-regression* | *10 or more in smallest subgroup; more than 15 in continuous meta-regression* | |
| Comment: Two trials compared. | | | | |
| **4: Was the direction of effect modification correctly hypothesized a priori?** | | | | |
| [ ] Definitely no | [**x**] Probably no or unclear | [ ] Probably yes | [ ] Definitely yes | |
| *Clearly post-hoc or results inconsistent with hypothesized direction or biologically very implausible* | *Vague hypothesis or hypothesized direction unclear* | *No prior protocol available but unequivocal statement of a priori hypothesis with correct direction of effect modification* | *Prior protocol available and includes correct specification of direction of effect modification, e.g., based on a biologic rationale* | |
| Comment: Authors did not hypothesize clear direction of each specific effect modification, only hypothesized overall direction. | | | | |
| **5: Does a test for interaction suggest that chance is an unlikely explanation of the apparent effect modification?** (consider irrespective of number of effect modifiers) | | | | |
| [**x**] Chance a very likely explanation | [ ] Chance a likely explanation or unclear | [ ] Chance may not explain | [ ] Chance an unlikely explanation | |
| *Interaction or meta-regression p-value >0.05* | *Interaction or meta-regression p-value ≤0.05 and >0.01, or no test of interaction reported and not computable* | *Interaction or meta-regression p-value ≤0.01 and >0.005* | *Interaction or meta-regression p-value ≤0.005* | |
| Comment: Subgroup of whether coronary heart disease or not had interaction p-value of 0.09. | | | | |
| **6: Did the authors test only a small number of effect modifiers or consider the number in their statistical analysis?** | | | | |
| [ ] Definitely no | [**x**] Probably no or unclear | [ ] Probably yes | [ ] Definitely yes | |
| *Explicitly exploratory analysis or large number of effect modifiers tested (e.g., greater than 10) and multiplicity not considered in analysis* | *No mention of number or 4-10 effect modifiers tested and number not considered in analysis* | *No protocol available but unequivocal statement of 3 or fewer effect modifiers tested* | *Protocol available and 3 or fewer effect modifiers tested or number considered in analysis* | |
| Comment: ETASS trial had seven effect modifiers and TESTS trial had eight effect modifiers. | | | | |
| **7: Did the authors use a random effects model?** | | | | |
| [ ] Definitely no | [ ] Probably no or unclear | [**x**] Probably yes | [ ] Definitely yes | |
| *Fixed (or common) effect or fixed effects model explicitly stated* | *Probably fixed effect(s) model* | *Probably random (or mixed) effects* | *Random (or mixed) effects explicitly stated* | |
| Comment: A model with random intercepts was used. | | | | |
| **8: If the effect modifier is a continuous variable, were arbitrary cut points avoided?** [**x**] not applicable: not continuous | | | | |
| [ ] Definitely no | [ ] Probably no or unclear | [ ] Probably yes | [ ] Definitely yes | |
| *Analysis based on exploratory cut point(s), e.g., picking cut point associated with highest interaction p-value* | *Analysis based on cut point(s) of unclear origin* | *Analysis based on pre-specified cut point(s), e.g., suggested by prior RCT* | *Analysis based on the full continuum, e.g., assuming a linear or logarithmic relationship* | |
| Comment: Coronary heart disease is a dichotomous variable. | | | | |
| **9 Optional: Are there any additional considerations that may increase or decrease credibility?** (manual section 3.9) [ ] not applicable | | | | |
|  | [ ] Yes, probably decrease | [**x**] Yes, probably increase | | |
| Comment: The two trials exhibited a low risk of bias, and thymosin α1 showed potential to enhance immune dysfunction in chronic conditions.   \| **10: How would you rate the overall credibility of the proposed effect modification?**  The overall rating should be driven by the items that decrease credibility. The following provides a sensible strategy:   - All responses definitely or probably decrease credibility or unclear 🡪 very low - Two or more responses definitely decrease credibility 🡪 maximum usually low even if all other responses satisfy credibility criteria - One response definitely decreases credibility 🡪 maximum usually moderate even if all other responses satisfy credibility criteria - Two responses probably decrease credibility 🡪 maximum usually moderate even if all other responses satisfy credibility criteria - No response options definitely or probably decrease credibility 🡪 high very likely   Place a mark on the continuous line (or type “x” in editable version) \| \| \| \| \|  \| \| --- \| --- \| --- \| --- \| --- \| --- \| \|  \|  \| \| \| \|  \| \|  \|  \| \| \| \|  \| \|  \|  \| \|  \|  \| \| \| \|  \| \|  \|  \| \| \| \|  \| \|  \| **Very low credibility** \| **Low credibility** \| **Moderate credibility** \| **High credibility** \|  \| \|  \|  \|  \|  \|  \|  \| \|  \| Minimal to no support for effect modification;  Use overall effect for each subgroup \| Some but insufficient support for effect modification;  Use overall effect for each subgroup but note remaining uncertainty \| Likely effect modification;  Use separate effects for each subgroup but note remaining uncertainty \| Very likely effect modification;  Use separate effects for each subgroup \|  \| \| Comment: Consistency across studies is unclear. Coronary heart disease may have a likely effect but there is insufficient support. \| \| \| \| \| \| | | | | |

1. **Credibility assessment for diabetes subgroup**

| **CREDIBILITY ASSESSMENT** | | | | |
| --- | --- | --- | --- | --- |
| **Essential preliminary considerations to define the possible effect modification of interest** | | | |  |
| State a single candidate effect modifier (e.g., age or comorbidity): Diabetes | | | |  |
| Was the effect modifier measured before or at randomization? [**x**] yes, continue [ ] no, stop here and refer to manual for further instructions | | | |  |
| State a single outcome and time-point (e.g., mortality at 1 year follow-up): Mortality at 28-day follow-up | | | |  |
| State a single effect measure (e.g., relative risk or risk difference): Hazard ratio | | | |  |
| **1: Is the analysis of effect modification based on comparison within rather than between trials?** | | | | |
| [ ] Completely between | [ ] Mostly between or unclear | [ ] Mostly within | [**x**] Completely within | |
| *Subgroup analysis or meta-regression comparing overall effects of each individual trial. This is typical for aggregate data meta-analysis.* | *Subgroup analysis or meta-regression with most information coming from overall effects, but some trials providing within-trial subgroup information* | *Most trials providing within-trial subgroup information; or individual participant data analysis that combines within and between trial information* | *All trials providing within-trial subgroup information or individual participant data; and the analysis separates within from between trial information, e.g., meta-analysis of interactions* | |
| Comment: We acquired within-trial subgroup information of ETASS and TESTS, the patients were summarized to study interactions. | | | | |
| **2: For within-trial comparisons, is the effect modification similar from trial to trial?** [ ] Not applicable: no or one within-RCT comparison | | | | |
| [ ] Definitely not similar | [ ] Probably not similar or unclear | [**x**] Mostly similar | [ ] Definitely similar | |
| *Effect modification reported for two or more trials and clearly different directions* | *Effect modification not reported for individual trials or too imprecise to tell* | *Effect modification reported for two or more trials, mostly similar in direction, but considerable differences in magnitude* | *Effect modification reported for two or more trials, similar in direction, only some differences in magnitude* | |
| Comment: The direction of effect modification was mostly similar between two trials. | | | | |
| **3: For between-trial comparisons, is the number of trials large?** [ ] Not applicable: no between RCT comparison | | | | |
| [**x**] Very small | [ ] Rather small or unclear | [ ] Rather large | [ ] Large | |
| *1 or 2 or in smallest subgroup; 5 or less in continuous meta-regression* | *3-4 in smallest subgroup; 6-10 in continuous meta-regression* | *5-9 in smallest subgroup; 11 to 15 in continuous meta-regression* | *10 or more in smallest subgroup; more than 15 in continuous meta-regression* | |
| Comment: Two trials compared. | | | | |
| **4: Was the direction of effect modification correctly hypothesized a priori?** | | | | |
| [ ] Definitely no | [**x**] Probably no or unclear | [ ] Probably yes | [ ] Definitely yes | |
| *Clearly post-hoc or results inconsistent with hypothesized direction or biologically very implausible* | *Vague hypothesis or hypothesized direction unclear* | *No prior protocol available but unequivocal statement of a priori hypothesis with correct direction of effect modification* | *Prior protocol available and includes correct specification of direction of effect modification, e.g., based on a biologic rationale* | |
| Comment: Authors did not hypothesize clear direction of each specific effect modification, only hypothesized overall direction. | | | | |
| **5: Does a test for interaction suggest that chance is an unlikely explanation of the apparent effect modification?** (consider irrespective of number of effect modifiers) | | | | |
| [**x**] Chance a very likely explanation | [ ] Chance a likely explanation or unclear | [ ] Chance may not explain | [ ] Chance an unlikely explanation | |
| *Interaction or meta-regression p-value >0.05* | *Interaction or meta-regression p-value ≤0.05 and >0.01, or no test of interaction reported and not computable* | *Interaction or meta-regression p-value ≤0.01 and >0.005* | *Interaction or meta-regression p-value ≤0.005* | |
| Comment: Subgroup of whether diabetes mellitus or not had interaction p-value of 0.08. | | | | |
| **6: Did the authors test only a small number of effect modifiers or consider the number in their statistical analysis?** | | | | |
| [ ] Definitely no | [**x**] Probably no or unclear | [ ] Probably yes | [ ] Definitely yes | |
| *Explicitly exploratory analysis or large number of effect modifiers tested (e.g., greater than 10) and multiplicity not considered in analysis* | *No mention of number or 4-10 effect modifiers tested and number not considered in analysis* | *No protocol available but unequivocal statement of 3 or fewer effect modifiers tested* | *Protocol available and 3 or fewer effect modifiers tested or number considered in analysis* | |
| Comment: ETASS trial had seven effect modifiers and TESTS trial had eight effect modifiers. | | | | |
| **7: Did the authors use a random effects model?** | | | | |
| [ ] Definitely no | [ ] Probably no or unclear | [**x**] Probably yes | [ ] Definitely yes | |
| *Fixed (or common) effect or fixed effects model explicitly stated* | *Probably fixed effect(s) model* | *Probably random (or mixed) effects* | *Random (or mixed) effects explicitly stated* | |
| Comment: A model with random intercepts was used. | | | | |
| **8: If the effect modifier is a continuous variable, were arbitrary cut points avoided?** [**x**] not applicable: not continuous | | | | |
| [ ] Definitely no | [ ] Probably no or unclear | [ ] Probably yes | [ ] Definitely yes | |
| *Analysis based on exploratory cut point(s), e.g., picking cut point associated with highest interaction p-value* | *Analysis based on cut point(s) of unclear origin* | *Analysis based on pre-specified cut point(s), e.g., suggested by prior RCT* | *Analysis based on the full continuum, e.g., assuming a linear or logarithmic relationship* | |
| Comment: Diabetes mellitus is a dichotomous variable. | | | | |
| **9 Optional: Are there any additional considerations that may increase or decrease credibility?** (manual section 3.9) [ ] not applicable | | | | |
|  | [ ] Yes, probably decrease | [**x**] Yes, probably increase | | |
| Comment: The two trials exhibited a low risk of bias, and thymosin α1 showed potential to enhance immune dysfunction in chronic conditions.   \| **10: How would you rate the overall credibility of the proposed effect modification?**  The overall rating should be driven by the items that decrease credibility. The following provides a sensible strategy:   - All responses definitely or probably decrease credibility or unclear 🡪 very low - Two or more responses definitely decrease credibility 🡪 maximum usually low even if all other responses satisfy credibility criteria - One response definitely decreases credibility 🡪 maximum usually moderate even if all other responses satisfy credibility criteria - Two responses probably decrease credibility 🡪 maximum usually moderate even if all other responses satisfy credibility criteria - No response options definitely or probably decrease credibility 🡪 high very likely   Place a mark on the continuous line (or type “x” in editable version) \| \| \| \| \|  \| \| --- \| --- \| --- \| --- \| --- \| --- \| \|  \|  \| \| \| \|  \| \|  \|  \| \| \| \|  \| \|  \|  \| \|  \|  \| \| \| \|  \| \|  \|  \| \| \| \|  \| \|  \| **Very low credibility** \| **Low credibility** \| **Moderate credibility** \| **High credibility** \|  \| \|  \|  \|  \|  \|  \|  \| \|  \| Minimal to no support for effect modification;  Use overall effect for each subgroup \| Some but insufficient support for effect modification;  Use overall effect for each subgroup but note remaining uncertainty \| Likely effect modification;  Use separate effects for each subgroup but note remaining uncertainty \| Very likely effect modification;  Use separate effects for each subgroup \|  \| \| Comment: Consistency across studies is unclear. Diabetes may have a likely effect but there is insufficient support. \| \| \| \| \| \| | | | | |

1. PRISMA 2020 checklist

| **Section and Topic** | **Item #** | **Checklist item** | **Location where item is reported** |
| --- | --- | --- | --- |
| **TITLE** | | |  |
| Title | 1 | Identify the report as a systematic review. | Page 1 |
| **ABSTRACT** | | |  |
| Abstract | 2 | See the PRISMA 2020 for Abstracts checklist. | Page 1-2 |
| **INTRODUCTION** | | |  |
| Rationale | 3 | Describe the rationale for the review in the context of existing knowledge. | Page 2-3 |
| Objectives | 4 | Provide an explicit statement of the objective(s) or question(s) the review addresses. | Page 3 |
| **METHODS** | | |  |
| Eligibility criteria | 5 | Specify the inclusion and exclusion criteria for the review and how studies were grouped for the syntheses. | Page 4 |
| Information sources | 6 | Specify all databases, registers, websites, organisations, reference lists and other sources searched or consulted to identify studies. Specify the date when each source was last searched or consulted. | Page 3-4 |
| Search strategy | 7 | Present the full search strategies for all databases, registers and websites, including any filters and limits used. | Page 3-4 and supplementary files |
| Selection process | 8 | Specify the methods used to decide whether a study met the inclusion criteria of the review, including how many reviewers screened each record and each report retrieved, whether they worked independently, and if applicable, details of automation tools used in the process. | Page 4-5 |
| Data collection process | 9 | Specify the methods used to collect data from reports, including how many reviewers collected data from each report, whether they worked independently, any processes for obtaining or confirming data from study investigators, and if applicable, details of automation tools used in the process. | Page 4-5 |
| Data items | 10a | List and define all outcomes for which data were sought. Specify whether all results that were compatible with each outcome domain in each study were sought (e.g. for all measures, time points, analyses), and if not, the methods used to decide which results to collect. | Page 4-5 |
|  | 10b | List and define all other variables for which data were sought (e.g. participant and intervention characteristics, funding sources). Describe any assumptions made about any missing or unclear information. | Page 5 |
| Study risk of bias assessment | 11 | Specify the methods used to assess risk of bias in the included studies, including details of the tool(s) used, how many reviewers assessed each study and whether they worked independently, and if applicable, details of automation tools used in the process. | Page 5 |
| Effect measures | 12 | Specify for each outcome the effect measure(s) (e.g. risk ratio, mean difference) used in the synthesis or presentation of results. | Page 5-6 |
| Synthesis methods | 13a | Describe the processes used to decide which studies were eligible for each synthesis (e.g. tabulating the study intervention characteristics and comparing against the planned groups for each synthesis (item #5)). | Page 4 |
|  | 13b | Describe any methods required to prepare the data for presentation or synthesis, such as handling of missing summary statistics, or data conversions. | Page 5-6 |
|  | 13c | Describe any methods used to tabulate or visually display results of individual studies and syntheses. | Page 5-6 |
|  | 13d | Describe any methods used to synthesize results and provide a rationale for the choice(s). If meta-analysis was performed, describe the model(s), method(s) to identify the presence and extent of statistical heterogeneity, and software package(s) used. | Page 6 |
|  | 13e | Describe any methods used to explore possible causes of heterogeneity among study results (e.g. subgroup analysis, meta-regression). | Page 6 |
|  | 13f | Describe any sensitivity analyses conducted to assess robustness of the synthesized results. | Page 5 |
| Reporting bias assessment | 14 | Describe any methods used to assess risk of bias due to missing results in a synthesis (arising from reporting biases). | Page 6-7 |
| Certainty assessment | 15 | Describe any methods used to assess certainty (or confidence) in the body of evidence for an outcome. | Page 6 |
| **RESULTS** | | |  |
| Study selection | 16a | Describe the results of the search and selection process, from the number of records identified in the search to the number of studies included in the review, ideally using a flow diagram. | Page 6-7 and figure 1 |
|  | 16b | Cite studies that might appear to meet the inclusion criteria, but which were excluded, and explain why they were excluded. | Page 7 |
| Study characteristics | 17 | Cite each included study and present its characteristics. | Page 7 and table 1 |
| Risk of bias in studies | 18 | Present assessments of risk of bias for each included study. | Page 7 and supplementary file |
| Results of individual studies | 19 | For all outcomes, present, for each study: (a) summary statistics for each group (where appropriate) and (b) an effect estimate and its precision (e.g. confidence/credible interval), ideally using structured tables or plots. | Table 1 and supplementary table S1 |
| Results of syntheses | 20a | For each synthesis, briefly summarise the characteristics and risk of bias among contributing studies. | Page 7 and supplementary figure S1 |
|  | 20b | Present results of all statistical syntheses conducted. If meta-analysis was done, present for each the summary estimate and its precision (e.g. confidence/credible interval) and measures of statistical heterogeneity. If comparing groups, describe the direction of the effect. | Page 7-8, figure 2 and supplementary figure S5-6 |
|  | 20c | Present results of all investigations of possible causes of heterogeneity among study results. | Page 8 |
|  | 20d | Present results of all sensitivity analyses conducted to assess the robustness of the synthesized results. | Page 7-8 |
| Reporting biases | 21 | Present assessments of risk of bias due to missing results (arising from reporting biases) for each synthesis assessed. | Page 7-8 and supplementary figure S4 |
| Certainty of evidence | 22 | Present assessments of certainty (or confidence) in the body of evidence for each outcome assessed. | Page 8 and supplementary ICEMAN reports |
| **DISCUSSION** | | |  |
| Discussion | 23a | Provide a general interpretation of the results in the context of other evidence. | Page 9-10 |
|  | 23b | Discuss any limitations of the evidence included in the review. | Page 11 |
|  | 23c | Discuss any limitations of the review processes used. | Page 11 |
|  | 23d | Discuss implications of the results for practice, policy, and future research. | Page 11 |
| **OTHER INFORMATION** | | |  |
| Registration and protocol | 24a | Provide registration information for the review, including register name and registration number, or state that the review was not registered. | Page 3 |
|  | 24b | Indicate where the review protocol can be accessed, or state that a protocol was not prepared. | Page 3 |
|  | 24c | Describe and explain any amendments to information provided at registration or in the protocol. | Page 3-4 |
| Support | 25 | Describe sources of financial or non-financial support for the review, and the role of the funders or sponsors in the review. | Funding statement |
| Competing interests | 26 | Declare any competing interests of review authors. | Conflict of interest |
| Availability of data, code and other materials | 27 | Report which of the following are publicly available and where they can be found: template data collection forms; data extracted from included studies; data used for all analyses; analytic code; any other materials used in the review. | Data availability statement |

*From:*  Page MJ, McKenzie JE, Bossuyt PM, Boutron I, Hoffmann TC, Mulrow CD, et al. The PRISMA 2020 statement: an updated guideline for reporting systematic reviews. BMJ 2021;372:n71. doi: 10.1136/bmj.n71
